# Sex-inducing effects toward planarians widely present among parasitic flatworms

**DOI:** 10.1016/j.isci.2022.105776

**Published:** 2022-12-08

**Authors:** Kiyono Sekii, Soichiro Miyashita, Kentaro Yamaguchi, Ikuma Saito, Yuria Saito, Sayaka Manta, Masaki Ishikawa, Miyu Narita, Taro Watanabe, Riku Ito, Mizuki Taguchi, Ryohei Furukawa, Aoi Ikeuchi, Kayoko Matsuo, Goro Kurita, Takashi Kumagai, Sho Shirakashi, Kazuo Ogawa, Kimitoshi Sakamoto, Ryo Koyanagi, Noriyuki Satoh, Mizuki Sasaki, Takanobu Maezawa, Madoka Ichikawa-Seki, Kazuya Kobayashi

**Affiliations:** 1 Department of Biology, Faculty of Agriculture and Life Science, Hirosaki University, Bunkyo-cho 3, Hirosaki, Aomori 036-8561, Japan; 2 Department of Biology, Research and Education Center for Natural Sciences, Keio University, 4-1-1 Hiyoshi, Kohoku-ku, Yokohama, Kanagawa 223-8521, Japan; 3 Laboratory of Veterinary Parasitology, Faculty of Agriculture, Iwate University, 3-18-8 Ueda, Morioka, Iwate 020-8550, Japan; 4 Faculty of Applied Biological Sciences, Gifu University, 1-1 Yanagido, Gifu 501-1193, Japan; 5 Kurita Animal Hospital, 139-1 Koga, Koga, Ibaraki 306-0016, Japan; 6 Section of Environmental Parasitology, Graduate School, Tokyo Medical & Dental University, 1-5-45, Yushima, Bunkyo-ku, Tokyo 113-8510, Japan; 7 Aquaculture Research Institute, Kindai University, Shirahama 3153, Nishimuro-gun, Wakayama 649-2211, Japan; 8 Meguro Parasitological Museum, 4-1-1 Shimo-Meguro, Meguro, Tokyo 153-0064, Japan; 9 Marine Genomics Unit, Okinawa Institute of Science and Technology Graduate University, Onna, Okinawa 904-0495, Japan; 10 Department of Parasitology, Asahikawa Medical University, 2-1-1-1 Midorigaoka-Higashi, Asahikawa, Hokkaido 078-8510, Japan; 11 Department of Integrated Science and Technology, National Institute of Technology, Tsuyama College, 624-1 Numa, Tsuyama, Okayama 708–8509, Japan

**Keywords:** Parasitology, Developmental genetics

## Abstract

Various parasitic flatworms infect vertebrates for sexual reproduction, often causing devastating diseases in their hosts. Consequently, flatworms are of great socioeconomic and biomedical importance. Although the cessation of parasitic flatworm sexual reproduction is a major target of anti-parasitic drug design, little is known regarding bioactive compounds controlling flatworm sexual maturation. Using the planarian *Dugesia ryukyuensis*, we observed that sex-inducing substances found in planarians are also widespread in parasitic flatworms, such as monogeneans and flukes (but not in tapeworms). Reverse-phase HPLC analysis revealed the sex-inducing substance(s) eluting around the tryptophan retention time in the fluke *Calicophoron calicophorum*, consistent with previous studies on the planarian *Bipalium nobile*, suggesting that the substance(s) is likely conserved among flatworms. Moreover, six of the 18 ovary-inducing substances identified via transcriptome and metabolome analyses are involved in purine metabolism. Our findings provide a basis for understanding and modifying the life cycles of various parasitic flatworms.

## Highlights

• Substances that can fully sexualize planarians are widespread in parasitic flatworms
• The sex-inducing substance is likely common among planarians and parasitic flatworms
• Eighteen ovary-inducers were identified, 6 of which are involved in purine metabolism
• Metabolome and transcriptome analyses support the importance of purine metabolism

## Graphical abstract

**Figure:**
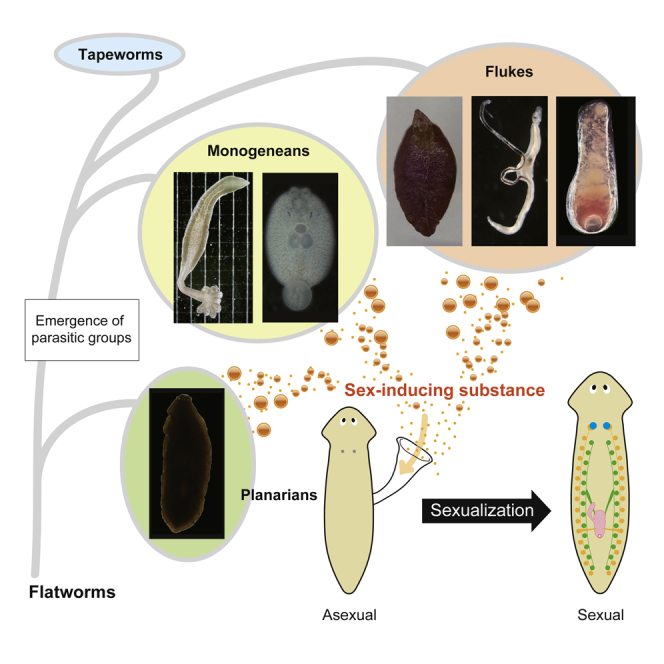

## Introduction

Parasitic flatworms infect many vertebrates to complete their sexual reproduction cycle. They spend most of their energy, which is obtained from the host, on egg production, often causing anemia, inflammation, and other problems in their hosts. Such host diseases can lead to substantial socioeconomic losses stemming from human health issues and reduced productivity in livestock and fish farming.^1^ Therefore, elucidating the mechanisms underlying the sexual reproduction of parasitic flatworms, especially the underlying bioactive compounds, is of particular economic and biomedical importance, as it could facilitate the design of anti-parasitic drugs that inhibit rapid asexual and harmful sexual reproduction in parasitic flatworms. However, little is known regarding these mechanisms, owing to the difficulty of maintaining the complicated flatworm life cycle in laboratory settings as well as of observing sexual maturation within the host.

**Figure 1:**
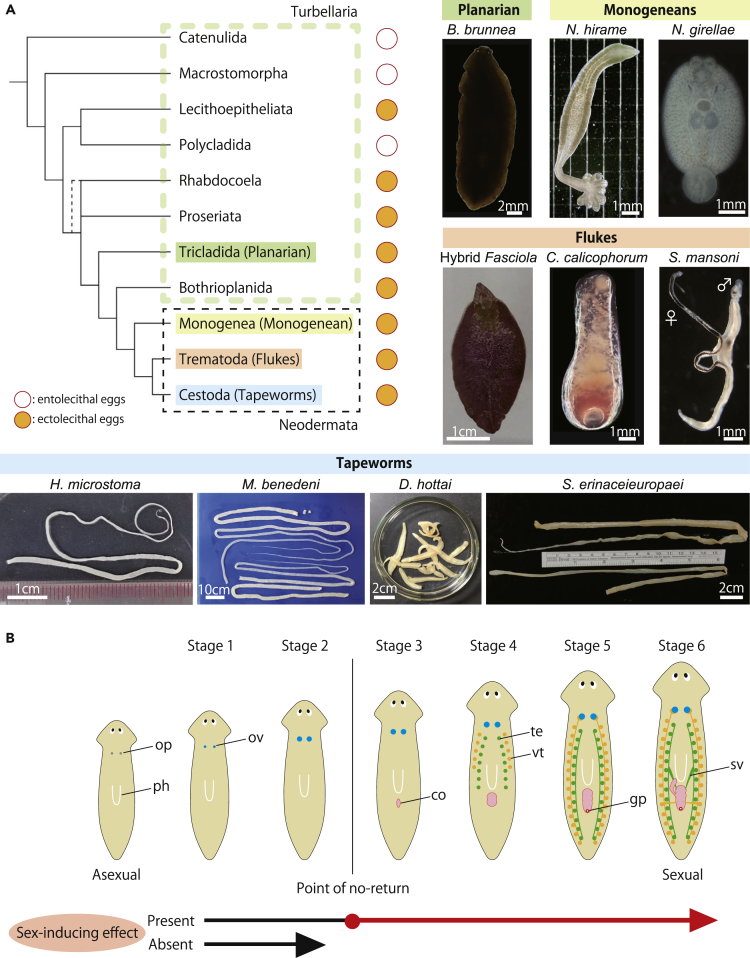
Flatworms in the present study (A) Major groups of flatworms and their phylogenetic relationships.^2^^,^^14^ Together with another group of flatworms (Bothrioplanida), the planarians (Tricladida) and parasitic flatworms (Neodermata) constitute a monophyletic group. Images are of the flatworm species used in the present study. *Bdellocephala brunnea* (class Turbellaria, order Tricladida, family Dendrocoelidae) was already known to harbor sex-inducing substances^11^^,^^15^ and, thus, was used as a positive control. Monogeneans (Monogenea): *Neoheterobothrium hirame* (order Mazocraeidea, family Diclidophoridae) and *Neobenedenia girellae* (order Capsalidea, family Capsalidae). Flukes (Trematoda): *Fasciola hepatica* × *Fasciola gigantica* hybrid (order Echinostomida, family Fasciolidae), *Calicophoron calicophorum* (order Plagiorchiida, family Paramphistomatidae), and *Schistosoma mansoni* (order Diplostomida, family Schistosomatidae). Tapeworms (Cestoda): *Hymenolepis microstoma* (order Cyclophyllidea, family Hymenolepididae), *Moniezia benedeni* (order Cyclophyllidea, family Anoplocephalidae), *Diphyllobothrium hottai* (order Pseudophyllidea, family Diphyllobothriidae), and *Spirometra erinaceieuropaei* (order Pseudophyllidea, family Diphyllobothriidae). (B) Six stages of sexualization in the planarian *Dugesia ryukyuensis*. The worm begins to develop hermaphroditic reproductive organs on switching from the asexual to the sexual state. Briefly, the asexual worm possesses the ovarian primordia (op); during stage 1, the number of oogonia increases in the ovary (ov); during stage 2, the ovary begins to mature and develop oocytes; during stage 3, the copulatory organ (co) begins to form; during stage 4, the primordial testis (te) and primordial vitellaria (vt)^16^ begin to form; during stage 5, the genital pore (gp) becomes externally apparent; during stage 6, the worm becomes sexually mature with well-developed seminal vesicles (sv), ready for mating and egg-laying. Between stages 2 and 3, there is a point of no-return, marking the developmental phase from which the sexualization process proceeds without further administration of sex-inducing substances. The sex-inducing effects were evaluated based on whether the OH strain of the planarian *D. ryukyuensis* changed beyond the point of no-return in a feeding bioassay. Note that the increase in planarian body size is due to the feeding bioassay procedure: if a food does not contain sex-inducing substances, asexual worms become bigger without gonadal development. Ph, pharynx.

Elucidating the common principles underpinning the sexual reproduction of parasitic flatworms would greatly enhance our understanding and ability to control a wide range of these species. In the present study, we focused on specific bioactive compounds, sex-inducing substances reported in planarians (nonparasitic flatworms). We hypothesized that these substances would be key factors in the common mechanisms of sexual reproduction if also present in parasitic flatworms. Planarians (order Tricladida) are the evolutionary ancestors of parasitic flatworms (Figure 1A), constituting a monophyletic group together with parasitic flatworms (Figure 1A, Neodermata) and another group of flatworms (Figure 1A, Bothrioplanida). Owing to the relative ease and safety of handling, planarians are emerging as excellent model organisms for studying their parasitic relatives.^2^^,^^3^ Like parasitic flatworms that combine both reproductive modes in their complex life cycle, some planarians can utilize asexual and sexual reproduction depending on environmental conditions. In planarians, the use of both reproductive modes is enabled by the presence of pluripotent stem cells called neoblasts,^4^^,^^5^^,^^6^ which can differentiate into any type of tissue required for a new individual(s) in the asexual mode or germ cells and other reproductive organs in the sexual mode. Grasso and Benazzi found that feeding asexual *Dugesia gonocephala s.l.* worms with sexual *Polycelis nigra* and *Dugesia lugubris* worms induced sexualization, clearly demonstrating that sexual planarians contain putative hormone-like chemicals (hereafter referred to as sex-inducing substances) that are not species-specific.^7^ Experimental sexualization by feeding is not a phenomenon restricted to *D. gonocephala s.l.*, but has been observed in several planarian species.^8^^,^^9^^,^^10^ Sex-inducing substances can induce sexual reproduction in asexual planarians by tuning the function and behavior of stem cells for sexual reproduction.^11^^,^^7^^,^^10^^,^^12^^,^^13^ Planarians that can switch reproductive modes achieve this by controlling the production of such substances in response to environmental and other factors. However, the presence and identity of sex-inducing substances in parasitic flatworms remain elusive. If sex-inducing substances are found in parasitic flatworms, this might imply that such substances are the trigger for their sexual maturation, which could be of major importance for future studies of parasitic worms.

The planarian *Dugesia ryukyuensis* (phylum Platyhelminthes, class Turbellaria, order Tricladida) is a particularly suitable model organism for studying sex-inducing substances. The OH strain of *D. ryukyuensis* is exclusively asexual, reproducing by transverse fission and subsequent regeneration. However, these flatworms can be experimentally and stably sexualized by feeding them sexual flatworms containing sex-inducing substances, which distinguishes *D. ryukyuensis* from other planarian species.^9^^,^^11^ This feeding assay system enables us to determine whether the species of interest possess the conserved sex-inducing substances and to examine the sex-inducing effects of specific chemical compounds.

There are two reasons to expect the presence of sex-inducing substances in parasitic flatworms. First, although the molecular identity of sex-inducing substances has not yet been determined, these are likely conserved across species, at least within the nonparasitic planarians of the order Tricladida,^15^ and the presence of sex-inducing substances in their cocoons containing numerous vitellocytes and several fertilized oocytes (i.e., ectolecithal eggs) has been reported. When the marine flatworm *Thysanozoon brocchii*, which does not have an ectolecithal egg system, and the slug *Ambigolimax valentianus* (non-flatworm tissue) were fed to *D. ryukyuensis*, none exerted a sex-inducing effect able to fully sexualize the worms.^15^ These results suggest that vitellocytes are important for the production and/or storage of sex-inducing substances and that sex-inducing substances are conserved within species with an ectolecithal egg system.^15^ Therefore, parasitic flatworms, which do have an ectolecithal egg system (Figure 1A), are expected to contain sex-inducing substances. Second, recent studies have revealed similarities in stem cell heterogeneity and gene expression signatures between flukes and nonparasitic planarians,^17^^,^^18^^,^^19^ and the complex life cycle of parasitic flatworms is considered to have evolved by adapting a developmental program already present in their nonparasitic ancestors.^2^^,^^20^ Therefore, a system regulating neoblasts via sex-inducing substances is also expected to be shared among parasitic flatworms.

Herein, we employed a *D. ryukyuensis* feeding assay system to elucidate whether the substances in different flatworms induce sexualization in *D. ryukyuensis*, with a focus on parasitic species, as well as to determine what constitutes sex-inducing substances in flatworms.

## Results

### The presence of sex-inducing effects in parasitic flatworms

Flatworms (phylum Platyhelminthes) consist of four major taxonomic groups: one nonparasitic group (Turbellaria) and its three parasitic evolutionary descendant groups, namely the monogeneans (Monogenea), flukes (Trematoda), and tapeworms (Cestoda) (Figure 1A). Monogeneans are ectoparasites that live on the gills or skin of aquatic vertebrates, whereas flukes and tapeworms live endoparasitically inside the digestive tracts, blood, or internal organs of aquatic and terrestrial vertebrates, often with a complex life cycle that involves host switching. To investigate the presence of sex-inducing effects among flatworms, we studied a wide variety of species (Figure 1A), including a species from the nonparasitic group and nine species from the three parasitic groups. The nonparasitic planarian *Bdellocephala brunnea*, which belongs to a different family than the planarian *D. ryukyuensis*, was used as a positive control. *B. brunnea* has already been shown to exhibit sex-inducing effects on *D. ryukyuensis* in feeding bioassays.^9^^,^^11^^,^^15^ The other investigated species included the monogeneans *Neoheterobothrium hirame* and *Neobenedenia girellae*, the flukes *Fasciola hepatica* × *Fasciola gigantica* hybrid (hereafter called “Hybrid *Fasciola*”),^21^ *Calicophoron calicophorum*, and *Schistosoma mansoni*, and the tapeworms *Hymenolepis microstoma*, *Moniezia benedeni*, *Diphyllobothrium hottai*, and *Spirometra erinaceieuropaei*.

Chemical substances contained in each flatworm were fractionated into three fractions (Fr.), M0, M10, and M100 (Figure S1) according to their hydrophobicity. The sex-inducing effects of these fractions on the asexual OH strain of the planarian *D. ryukyuensis* were examined through 4 weeks of feeding bioassays. The sexualization process of the asexual OH worms can be roughly divided into six morphologically distinct stages (Figure 1B).^22^ During stages one and two, the development of ovaries occurs, whereas other reproductive organs form during stages 3–6.^9^^,^^16^^,^^22^ Between the second and third stages, there is a point of no-return that marks the capacity of the worm to autonomously complete the sexualization process without further administration of sex-inducing substances. Here, we define sex-inducing substances as those capable of making the asexual OH worms go beyond the point of no-return, and the sex-inducing effects were evaluated based on whether the tested *D. ryukyuensis* worms developed copulatory organs (Figure 1B). A full sex-inducing effect was defined as when the worm reached the point of no-return and the copulatory organ was observed. When only ovary formation was induced but the worm did not reach the point of no-return, this was termed an ovary-inducing effect.

**Figure 2:**
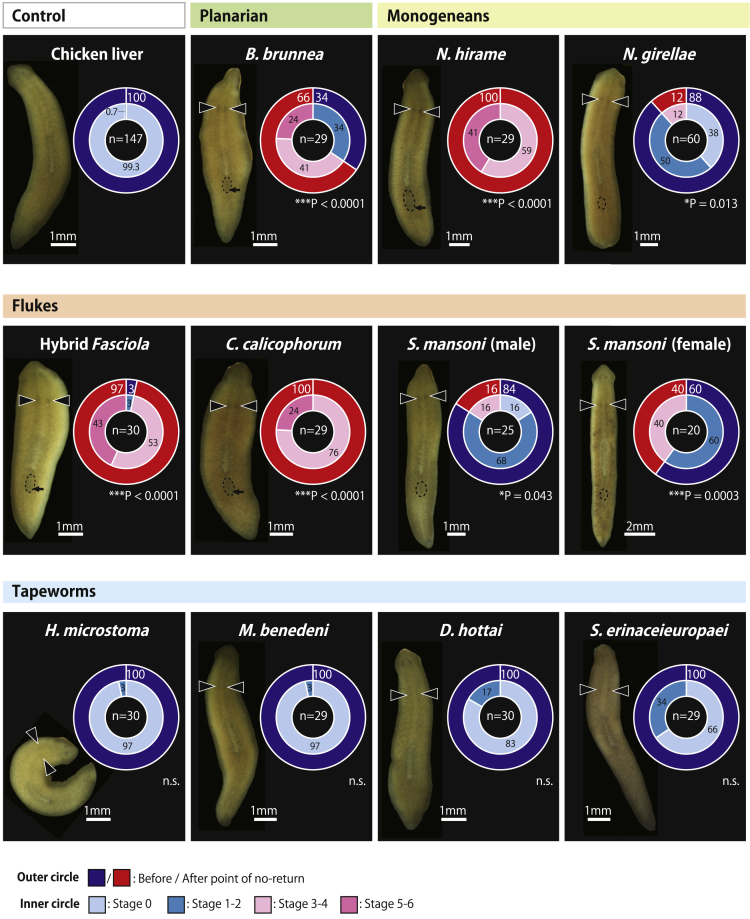
Sex-inducing effects of Fr. M10 from each flatworm species on asexual *Dugesia ryukyuensis* worms The results after 4 weeks of feeding bioassays. Note that the fluke *S. mansoni* females were fed directly without fractionation owing to collection difficulties associated with their small size. Images of the most sexually developed worms from each group are presented. Arrowheads indicate induced ovaries, dotted circles indicate copulatory organs, and arrows indicate genital pores. The percentages of worms are presented in doughnut charts: the outer circle shows the worms before and after the point of no-return, and the inner circle shows the sexualization stages observed in the worms. White and black numbers in the circles indicate percentages. Asterisks indicate significant differences in the number of worms before and after the point of no-return between the control and focal groups (Fisher’s exact test: ∗p< 0.05; ∗∗p< 0.01; ∗∗∗p< 0.001; n.s., not significant). Source data and statistics, including the exact p-values, are available in Dataset S5. The sample size of each group is shown in the center of the doughnut chart. The number of control worms is the sum of the control worms from several different bioassay batches.

**Figure 3:**
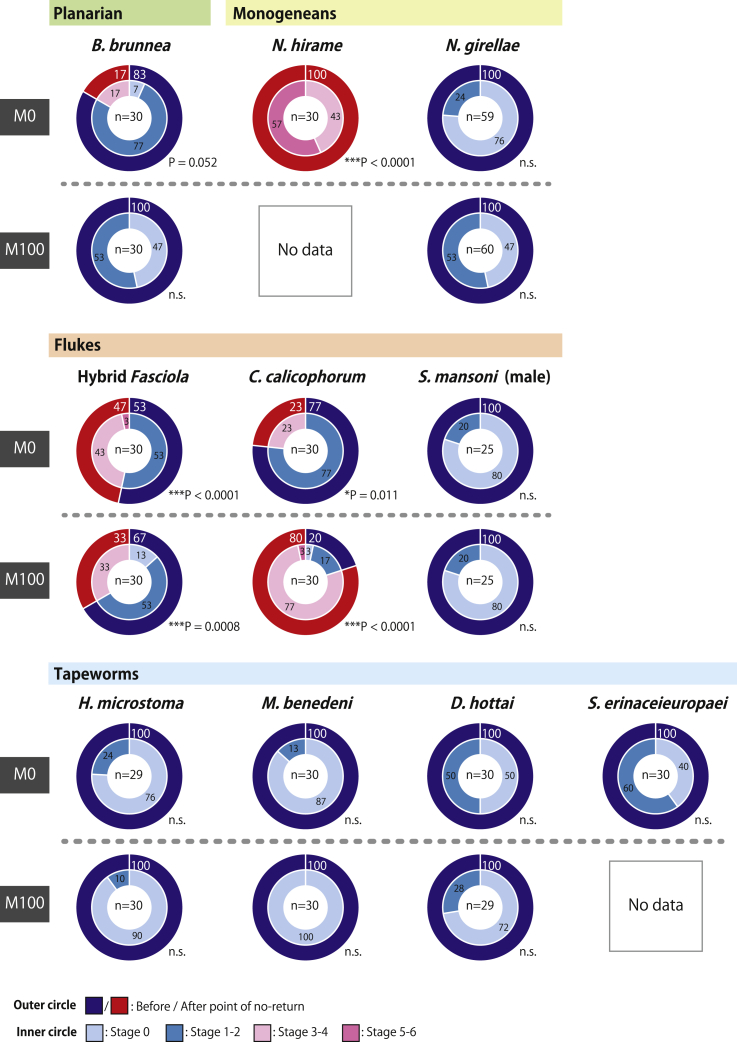
Sex-inducing effects of Fr. M0 and Fr. M100 from each flatworm species on asexual *Dugesia ryukyuensis* worms The results after 4 weeks of feeding bioassays. The percentages of worms in different developmental states are presented in doughnut charts: the outer circle shows the worms before and after the point of no-return, and the inner circle shows the sexualization stages of worms. White and black numbers in the circles indicate percentage values. Asterisks indicate significant differences in the number of worms before and after the point of no-return between the control and focal groups (Fisher’s exact test: ∗p< 0.05; ∗∗p< 0.01; ∗∗∗p< 0.001; n.s., not significant). Source data and statistics, including the exact p-values, are available in Dataset S5. The sample size of each group is shown in the center of the doughnut chart. The results for Fr. M100 of the monogenean *N. hirame* and the tapeworm *S. erinaceieuropaei* are not available because the test worms did not eat in the feeding bioassays. Thus, it was impossible to evaluate the sex-inducing effects of Fr. M100 of these species upon*D. ryukyuensis*.

Using this definition, we observed full sex-inducing effects in all examined species from the monogenean and fluke groups as well as the planarian *B. brunnea* (Figure 2). Consistent with the results of a previous study on planarian *B. brunnea*,^15^ Fr. M10 consistently exhibited full sex-inducing effects among the three fractions, with the most potent effects observed in six out of seven species (Figures 2 and 3). With regards to the fluke *S. mansoni*, males and females were examined separately. This fluke, or rather the family Schistosomatidae to which it belongs, is gonochoristic (i.e., individuals are either male or female), which is unusual for flatworms as most species are hermaphrodites.^2^ Notably, full sex-inducing effects were observed in both sexes (Figure 2), although females were directly fed without fractionation owing to collection difficulties associated with their small size. On the other hand, none of the tapeworms exhibited full sex-inducing effects in any of the three fractions, although showing partial sex-inducing effects with ovary formation induced in some test worms (Figures 2 and 3).

In addition to the Fr. M10, the Fr. M100 of the flukes Hybrid *Fasciola* and *C. calicophorum* exhibited full sex-inducing effects (Figure 3), which was not the case for the planarian *B. brunnea* or any other planarians examined in a previous study.^15^ This may be explained by the excessive amounts of sex-inducing substances that were not eluted in Fr. M10 and, thus, carried over to Fr. M100, suggesting the conservation of sex-inducing substances. Alternatively, different substances (highly hydrophobic substances in Fr. M100) may work as sex-inducing substances in the flukes. To discriminate between these two possibilities, we performed an additional experiment using the fluke *C. calicophorum*, wherein five fractions were produced, namely Fr. M0, Fr. M10, Fr. M30, Fr. M50, and Fr. M100 (Figure S2A). The strongest sex-inducing effect was found in Fr. M10 (FigureS2B), which induced copulatory organ development in all worms (overcoming the point of no-return). Moreover, the previously observed full sex-inducing effect of Fr. M100 shifted to Fr. M30 (Figure S2B). Therefore, the explanation for full sex-inducing effects previously exhibited by Fr. M100 seems to be a carryover of the same chemical compounds in Fr. M10. Although the possibility that the fluke *C. calicophorum* might possess slightly different hydrophobic sex-inducing substances that are inevitably eluted in Fr. M30 cannot be completely excluded, overall results suggest that the same chemical compounds in Fr. M10 are primarily responsible for full sex-inducing effects among flatworms, implying the conservation of sex-inducing substances.

**Figure 4:**
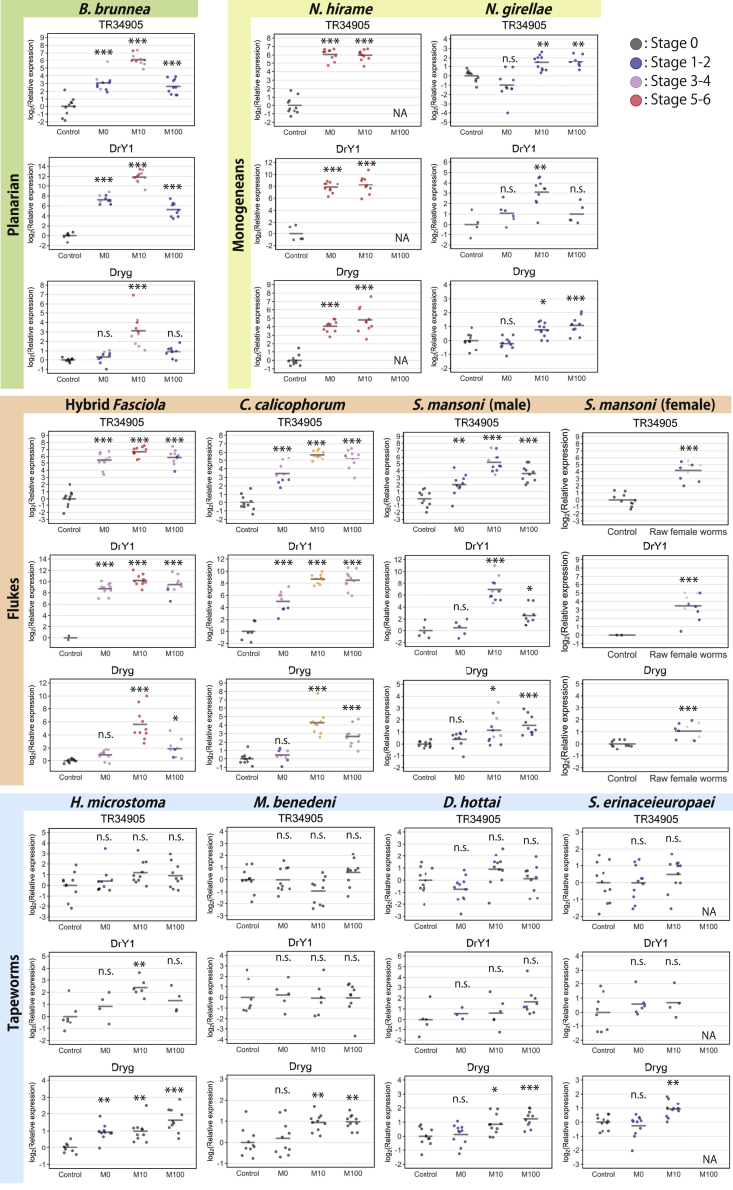
qRT-PCR analysis to examine the induction of reproductive organs The expression levels of the ovary, testis, and vitellaria marker genes (*TR34905|c0_g1_i1*, *DrY1*, and *Dryg*, respectively) were examined in worms following 4 weeks of feeding bioassays. qRT-PCR data for the Fr. M0-, M10-, and M100-fed groups are presented relative to the expression level in the control worms, and log_2_ (relative expression) on the vertical axis indicates 2^–ΔΔCt^. Each circle represents an individual worm. From each group, the 10 or 11 most sexually developed worms were selected for qRT-PCR analysis. Data (circles) are not shown if the expression was too low to be detected (handled as not available [N.A.]). Bars in the plots indicate average 2^–ΔΔCt^ values. Asterisks indicate significant differences between the control group and the Fr. M0-, M10-, and M100-fed groups (Tukey’s honestly significant difference [HSD] test: ∗p< 0.05; ∗∗p< 0.01; ∗∗∗p< 0.001; n.s., not significant). Source data and statistics, including the exact p-values, are available in Dataset S5. Note that *DrY1* and *Dryg* were detected in the control (asexual) group owing to the high sensitivity of qRT-PCR. However, neither testes nor vitellaria were morphologically observed in asexual worms. The circle color corresponds to the observed morphological changes. Data shown in orange denote worms for which information on morphology was accidentally lost during the RNA extraction process as a result of a laboratory labeling error.

The expression of markers for reproductive organs was examined by quantitative reverse transcription polymerase chain reaction (qRT-PCR) because the testes and vitellaria are not visible under a microscope. In this analysis, the testis and vitellaria marker genes *DrY1*^12^^,^^23^^,^^24^ and *Dryg*,^16^ respectively, were used. An ovary marker gene has not been described for the planarian *D. ryukyuensis*, although reported in the planarian *Schmidtea mediterranea*.^25^^,^^26^ In the present study, we successfully isolated *TR34905|c0_g1_i1* as an ovary marker gene (Figure S3). Using these three marker genes, we examined 10 or 11 most sexually developed worms from each feeding bioassay group via qRT-PCR. Consistent with our microscopic observations, the worms fed Fr. M10 from species with full sex-inducing effects (i.e., the planarian *B. brunnea*, monogeneans, and flukes) showed a significantly upregulated expression for ovary, testis, and vitellaria marker genes when compared to control group worms (Figure 4). These results support the notion that the slightly hydrophobic chemical compounds in Fr. M10 are responsible for full sex-inducing effects in the parasitic flatworms and the planarian *B. brunnea*.

Intriguingly, in certain tapeworm-fed groups, the testis and vitellaria marker genes were occasionally upregulated (Figure 4). However, when anatomically analyzed by sectioning (Figure S4), organized tissue structures for these organs were not observed in most cases. The only exception was the observation of small testes in the worm treated with Fr. M10 of the tapeworm *H. microstoma*, which is consistent with significantly increased testis marker gene expression. However, the observed testes were only two in the whole body, and the size was very small compared to those of worms treated with Fr. M10 of the planarian *B. brunnea*, with no spermatozoa or sperm cells observed. Therefore, the effect of *H. microstoma* Fr. M10 in inducing organized testes seemed to be weak. Copulatory organs were absent in the observed sections. Ovary marker gene expression in tapeworm-fed groups was never significantly different from that in control worms (Figure 4). These results suggest a failure in the first important step, which was also supported by the small ovaries observed in tissue sections (Figure S4), that is, the ovaries had not developed sufficiently enough to allow sexualization to proceed properly.

### Sex-inducing substances likely conserved among planarians and parasitic flatworms

Sex-inducing substances were present in the planarians, monogeneans, and flukes, but without structural estimation, such as through NMR, it cannot be concluded whether the responsible substances are the same. We, therefore, established a new purification method for sex-inducing substances, which overcomes the problem of conventional approaches where the fraction with sex-inducing activity contains a large amount of tryptophan.^22^^,^^27^ First, a new large-scale purification method via open-column chromatography (Figure S5A) was established using the planarian *Bipalium nobile*, which has a large body size, easily ensuring the required amount of starting material for this method. With this method, the full sex-inducing effects observed in Fr. M10 with the previous method using Sep-Pak cartridges were shifted to Fr. M30 (Figure S5B). When this method was applied to the fluke *C. calicophorum*, the full sex-inducing effects were similarly shifted to Fr. M30 (Figure S5B). The advantage of this method was that tryptophan, which is abundant in sexual flatworms, was mainly retained in Fr.M10 and less in Fr.M30 (Figure S5C).

**Figure 5:**
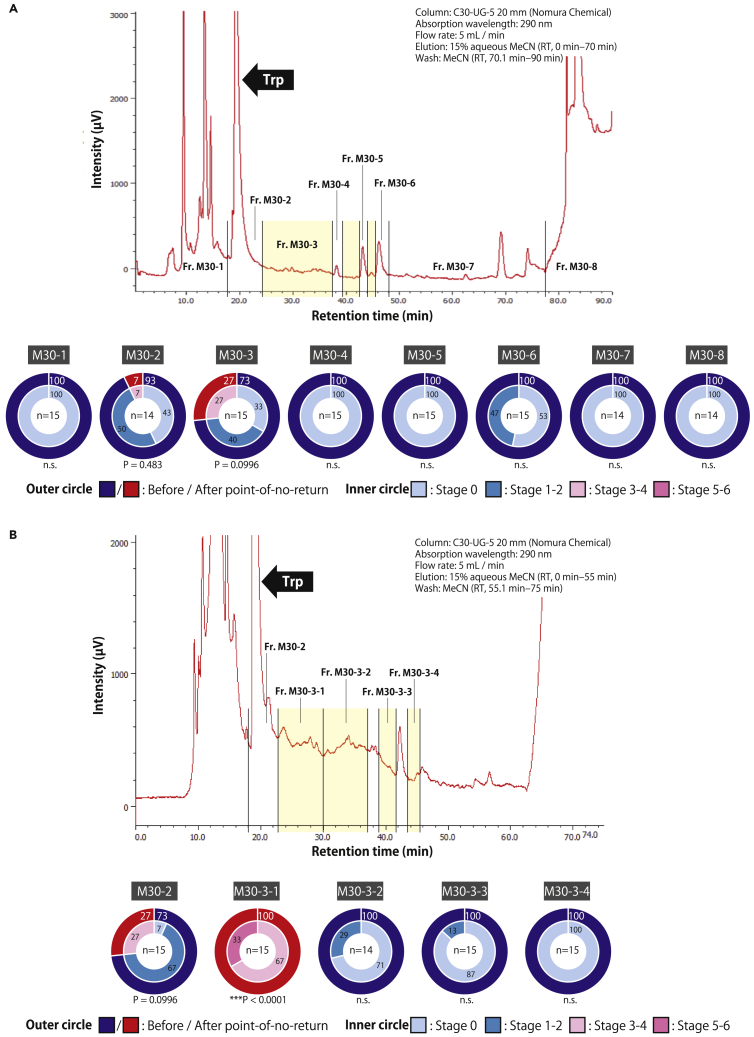
Sex-inducing substances eluted immediately after tryptophan (Trp) in reverse-phase HPLC (A) Fr. M30 of the fluke *C. calicophorum*, which was obtained through a new fractionation method using open-column chromatography, was further fractionated into eight fractions via reverse-phase HPLC. Yellow indicates Fr. M30-3, which was collected from several areas that showed no apparent peak. The samples derived from 2 g of *C. calicophorum* worms were injected into the HPLC system via four separate injections. (B) Fr. M30-3 indicated in yellow were further fractionated into four fractions via reverse-phase HPLC. Note that several peaks were observed in Frs. M30-3-1 and M30-3-2, but these peaks did not appear every time. Therefore, Frs. M30-3-1 and M30-3-2 were collected based on the retention time (approximately every 7 min) after the appearance of the tryptophan peak. The samples derived from 2 g of *C. calicophorum* worms were injected into the HPLC system via four separate injections. The sex-inducing effects of each fraction on asexual *Dugesia ryukyuensis* worms were examined using a feeding bioassay for 4 weeks. The percentages of worms in different developmental states are presented in doughnut charts; the outer circle shows the worms before and after the point of no-return, and the inner circle shows the sexualization stages of the worms. White and black numbers in the circles indicate percentages. Asterisks indicate significant differences in the number of worms before and after the point of no-return between the control and focal groups (Fisher’s exact test: ∗∗∗p< 0.001; n.s., not significant). Source data and statistics, including the exact p-values, are available in Dataset S5. The sample size of each group is shown in the center of the doughnut chart.

Fr. M30 of *C. calicophorum* was then subjected to reverse-phase high-performance liquid chromatography (HPLC) fractionation, and the sex-inducing effect of conspicuous peaks and other fractions was examined. We found that Fr. M30-2 containing the tryptophan peak, and Fr. M30-3, combining fractions without conspicuous peaks, exhibited full sex-inducing effects, although the efficacies did not significantly differ from the control group (Figure 5A). When the Fr. M30-3 was further fractionated and examined for sex-inducing effects, a significantly strong effect was found in Fr. M30-3-1, which eluted immediately after the tryptophan peak (Figure 5B). For Fr. M30 of *B. nobile*, further fractionations via reverse-phase HPLC were not possible because of the limited availability of starting materials and the instability of sex-inducing activity, as the worms need to be collected from wild environments while in their breeding season. However, the results for *C. calicophorum* were consistent with a previous study on *B. nobile* that demonstrated the presence of a substance different from tryptophan with sex-inducing activity near the retention time of tryptophan.^22^ There was no constantly detectable peak in Fr. M30-3-1 (Figures 5A and 5B), and the quantity of the responsible substance was not sufficient for structural estimation at this stage. However, a shift of the full sex-inducing effect to Fr. M30 under a new method, together with HPLC data from the present study and Kitamura et al.,^22^ collectively suggest that the substance responsible for full sex-inducing effects in *B. nobile* and *C. calicophorum* is likely the same compound eluting immediately after the tryptophan retention time in reverse-phase HPLC.

### Purine metabolism as the key factor in sex-inducing effects

The isolation and identification of sex-inducing substances were not yet achieved, but we considered that the substances could be known compounds. To obtain clues about the molecular identity of sex-inducing substances, we employed another approach. Our feeding bioassay (Figure 2) results revealed that parasitic flatworms with an ectolecithal egg system, excluding tapeworms, exhibited full sex-inducing effects. This is consistent with a previous study suggesting that vitellocytes are important for the production and/or storage of sex-inducing substances, and that the presence of such substances is conserved within species with ectolecithal egg systems.^15^ Therefore, we aimed to identify the sex-inducing substances present in vitellocytes and, hence, performed transcriptome (Figure S6) and metabolome (Figure S7) analyses of planarian *D. ryukyuensis* cocoons and sexual worms, both of which are enriched with vitellocytes.

**Figure 6:**
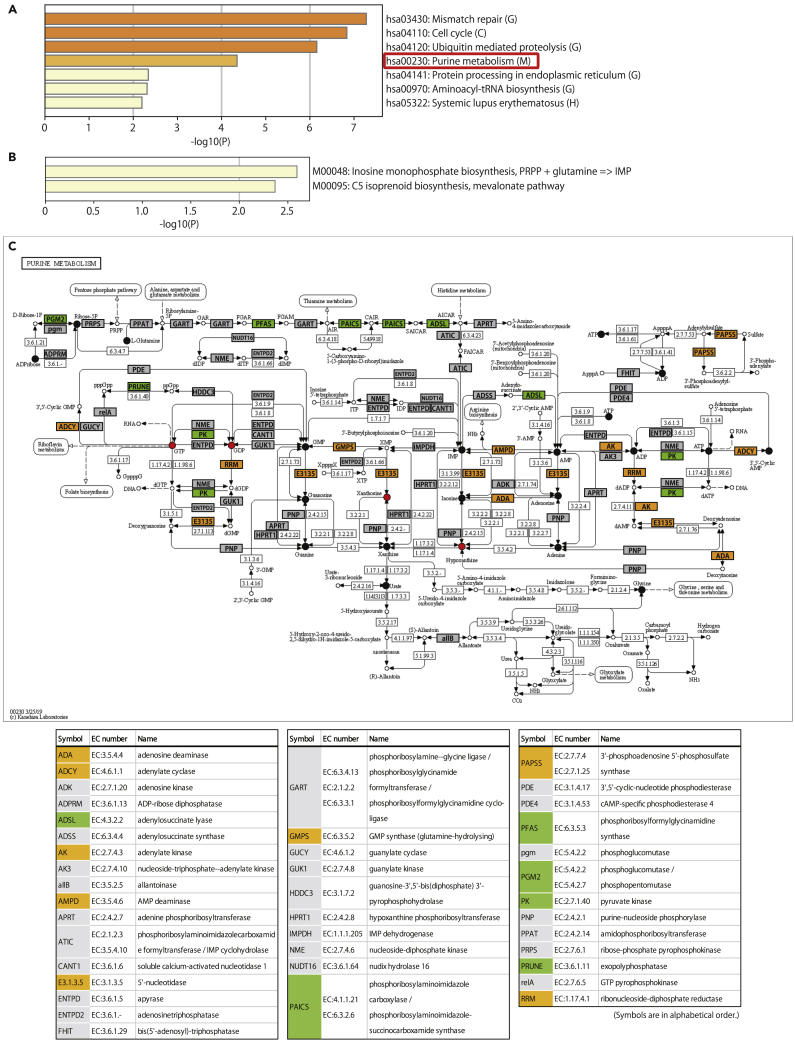
Transcriptome and metabolome analyses identified purine metabolism as a key pathway associated with sex-inducing effects (A) KEGG pathway enrichment analysis of Set 1 genes (sexual DEGs expressed in the planarian *Dugesia ryukyuensis* cocoons). Letters in brackets indicate categories in KEGG: M, “Metabolism;” G, “Genetic Information Processing;” C, “Cellular Processes;” H, “Human Diseases.” (B) KEGG pathway enrichment analysis of Set 2 genes, which were a subset of Set 1 genes showing high similarity with the contigs observed in planarian *Bdellocephala brunnea* cocoons. Statistically significant enriched pathways are presented with pathway ID in the KEGG database in descending order of –log_10_[p-value (*P*)]. Each bar is colored depending on the value of –log_10_ (P). (C) KEGG pathway mapping of purine metabolism. Rectangular orange boxes represent genes from the Set 1 group; green boxes represent genes from the Set 2 group; gray boxes represent genes expressed in the planarian transcriptome.^24^ Red circles represent metabolites present in the sexual worms and/or cocoons at a concentration five times higher than that observed in asexual worms (Table S1); black circles represent other metabolites detected in the metabolome analysis; white circles represent metabolites with no data available. Note that pyrophosphate and 3′-5′-cyclic dAMP are not specifically indicated in the figure because pyrophosphate is involved not only in purine metabolism but also in many other metabolic pathways, and 3′-5′-cyclic dAMP is not registered in KEGG purine metabolism.

First, RNA-sequencing (RNA-seq) of the planarian *D. ryukyuensis* cocoons was performed (Figure S6), and the *de novo* assembly of a transcript model was generated (Dataset S1). By combining this cocoon RNA-seq data with existing transcriptome datasets of adult sexual worms,^24^ the sexually biased differentially expressed genes (DEGs) present in the cocoons were selected as the first set of genes (Set 1; Dataset S2). These are candidate genes potentially involved in the production of sex-inducing substances in vitellocytes. Kyoto Encyclopedia of Genes and Genomes (KEGG)^28^ pathway enrichment analysis of Set 1 genes revealed that purine metabolism was the only enriched pathway identified for the “Metabolism” category, whereas all other enriched pathways were from non-metabolic categories such as “Genetic Information Processing,” “Cellular Processes,” and “Human Diseases” (Figure 6A). Furthermore, RNA-seq of the planarian *B. brunnea* cocoons was performed to investigate the presence of conserved enzymes between the planarians *D. ryukyuensis* and *B. brunnea* as we expected the enzymes involved in sex-inducing substance production to be relatively conserved. Using a *de novo* assembly of the planarian *B. brunnea* cocoon transcriptome model (Dataset S3), the second set of genes (Set 2) was derived from Set 1 (Dataset S4); these were highly similar to the contigs expressed in the planarian *B. brunnea* cocoon (e-value cutoff of e−30). KEGG pathway enrichment analysis of Set 2 revealed the enrichment of “inosine monophosphate (IMP) biosynthesis,” which plays a role in purine metabolism, and the “C5 isoprenoid biosynthesis, mevalonate pathway” (Figure 6B). Based on these findings, the genes in Sets 1 and 2 were mapped to purine metabolism in the KEGG pathway (Figure 6C, indicated in orange and green, respectively), revealing that the Set 1 and 2 genes were involved in producing primary purine metabolites such as GTP and ATP. Purine nucleotides can be synthesized via the *de novo* pathway or recycled from free bases via the salvage pathway. Set 2 genes mapped to the purine metabolism pathway were often found in the *de novo* purine nucleotide biosynthesis pathway (Figure 6C; indicated in green), wherein IMP, a purine nucleotide precursor, is synthesized from 5-phosphoribosyl-1-pyrophosphate (PRPP). Moreover, pyruvate kinase, which catalyzes the reaction GTP (ATP) + pyruvate ↔ GDP (ADP) + phosphoenolpyruvate (“Enzyme Commission [EC] 2.7.1.40” in Figure 6C), was also present among the Set 2 genes. These results suggest that purine-related metabolites are actively synthesized in planarian *D. ryukyuensis* cocoons, a feature that is likely conserved in the planarian *B. brunnea*.

**Figure 7:**
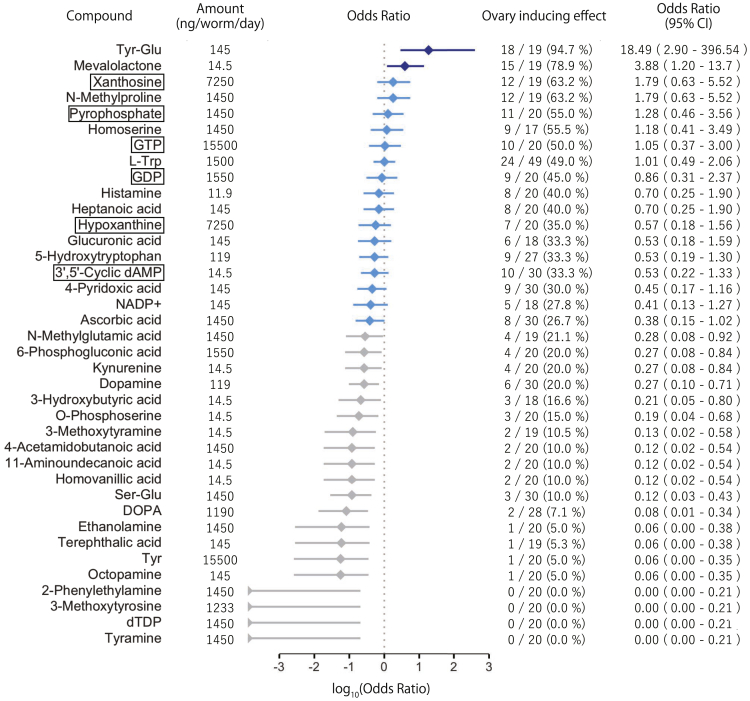
Ovary-inducing effects of candidate sex-inducing substances identified in the metabolomics analysis For each metabolite, the most-advanced sexualization results in the feeding bioassays are shown. The ovary-inducing effect of each metabolite was calculated by dividing the number of individuals with developing ovaries by the total number of individuals. The odds ratios were calculated in comparison with d-tryptophan (d-Trp), which was reported as an ovary-inducing substance in a previous study by Kobayashi et al.^27^ with an ovary-inducing effect of 48.8% (41/48). The results are presented in descending order of the odds ratio. Those with a lower 95% confidential interval (CI) > 1 are shown in blue; those with 95% CI across 1 are shown in light blue; and those with an upper 95% CI < 1 are shown in gray. Note that the results of d-Trp and l-tryptophan (l-Trp) used in the present study are from Kobayashi et al.^27^ Source data, statistics, and the other amounts examined in the feeding bioassays of each metabolite are available in Dataset S5. Metabolites enclosed in squares indicate those associated with purine metabolism.

Next, the metabolomes of *D. ryukyuensis* asexual worms, sexual worms, and cocoons were compared (Figure S7). Because the latter two exhibit full sex-inducing effects, we selected metabolites that were expressed much more highly (>5:1 ratio) in sexual worms and/or cocoons versus asexual worms (Table S1; 40 out of 228 metabolites) as potential sex-inducing substances. Note that the number of replicates used in the metabolome analysis was n = 1, so no statistical analysis was applied for comparison and selection. The sex-inducing effects of these candidates were examined through feeding bioassays using several different dosages (Dataset S5). Eighteen out of 38 tested metabolites exhibited ovary-inducing effects (partial sex-inducing effects) equivalent to that of d-Trp (Figure 7), which was previously identified as an ovary-inducing substance.^27^ The identified 18 ovary-inducing substances include six purine metabolism-associated substances, namely xanthosine, pyrophosphate, GTP, GDP, hypoxanthine, and 3′-5′-cyclic dAMP (Figure 6C, indicated by the red circle; Figure 7, indicated by the rectangle box). In the groups fed with four out of the six metabolites, namely xanthosine, pyrophosphate, GTP, and GDP, some test worms very rarely developed past the point of no-return (5–11%), but their efficacy was not significantly different from that of the control groups (Figure S8). Effective substances with “full” sex-inducing effects were not found. Yet, the results of transcriptome and metabolome analyses were reasonably consistent, strongly supporting the view that purine metabolism, including the six metabolites upregulated by the activation of genes in Sets 1 and 2, indeed play an important role in flatworm sexual maturation.

## Discussion

In our study, the full sex-inducing effects on *D. ryukyuensis* were widely observed among both nonparasitic and parasitic flatworms with ectolecithal eggs (i.e., a cocoon containing oocytes and vitellocytes), namely the planarians (nonparasitic), monogeneans (ectoparasitic), and flukes (endoparasitic). New fractionation methods using open-column chromatography and reverse-phase HPLC have also revealed that the substance(s) responsible for full sex-inducing effects contained in the fluke *C. calicophorum* is eluted near the retention time of tryptophan, consistent with previous results for the planarian *B. nobile*.^22^ These results indicate that the substance(s) responsible for the full sex-inducing effects is likely conserved among flatworms, despite their phylogenetic distances and distinct lifestyles. Moreover, we identified that purine metabolism plays a key role in flatworm sexualization, as six out of 18 identified ovary-inducing substances were associated with purine metabolism.

Notably, both male and female *S. mansoni* worms (fluke) exhibited full sex-inducing effects. These results were unexpected because our previous study showed that sex-inducing substances are possibly contained in vitellocytes,^15^ which are not present in male *S. mansoni*. However, the current results are reasonable, assuming that sex-inducing substances are necessary for the maintenance of both male and female sexuality. The most likely explanation is that males obtained sex-inducing substances from females with vitellaria because the samples for feeding bioassays were prepared by collecting sexually mature paired worms of the fluke *S. mansoni* and then separating them into males and females. Consistently, the full sex-inducing effects of Fr. M10 from *S. mansoni* males were moderate compared to those of flukes Hybrid *Fasciola* and *C. calicophorum* (Figure 2). These two fluke species possess numerous vitelline glands throughout their bodies and thus probably contain large amounts of sex-inducing substances, as indicated by the finding that Fr. M10 exhibited strong sex-inducing effects with 97 and 100% of the test worms passing the point of no-return, respectively (Figure 2). After pairing, *S. mansoni* male and female stay attached forever, which is akin to a “functional” hermaphrodite.^2^ However, the dry weights of females are approximately 3.8 times smaller than those of males.^29^ The lower amount of sex-inducing substances in *S. mansoni* males compared to the other two flukes could reflect that *S. mansoni* males may primarily receive the sex-inducing substances from females. Alternatively, it is possible that *S. mansoni* males also produce sex-inducing substances. Of interest, males occasionally develop pseudo-ovaries and vitellaria in addition to the testis.^30^^,^^31^^,^^32^ Thus, *S. mansoni* males may possess the cells responsible for the production of sex-inducing substances as vestigial remnants originating from the vitellaria of their hermaphroditic ancestors. In most *Schistosoma* species (flukes), the females are incapable of reaching sexual maturity until paired with males, whereas males can develop the testes to some extent in the absence of females.^33^^,^^34^ Although the chemical factors acting on the surface of physical contact have been enigmatic for a long time, our study raises the possibility that sex-inducing substances produced by males may be one such factor.

Tapeworms also possess an ectolecithal egg system. Intriguingly, however, no tapeworms in our study exhibited full sex-inducing effects, only ovary induction. One possible explanation for this is the reduced size and function of tapeworm vitellaria compared to that of flukes,^35^ which may result in low levels of sex-inducing substances. A previous study showed that full sex-inducing effects of the planarian *B. brunnea* Fr. M0 + M10 (i.e., the combined fraction) drastically declined after a 5-fold dilution.^36^ Therefore, sex-inducing substances in tapeworms in the quantities used herein may not have been sufficient to exert full sex-inducing effects in the planarian *D. ryukyuensis*. However, the other most likely explanation is that the chemical compound sets required for sexual maturation in tapeworms may be slightly different. This possibility is supported by feeding bioassay and qRT-PCR results, wherein marker gene expression for reproductive organs developing after the point of no-return (i.e., testis and vitellaria) sometimes increased unexpectedly even though the marker gene expression for ovary development (the first important step of sexualization) never increased. Indeed, when tissue sections were examined, very small testes were observed, albeit exceptionally, in a group fed with Fr. M10 of *H. microstoma*. In the planarian *S. mediterranea*, cocoon production is possible in the absence of gametes, mating, and fertilization.^37^ In the case of *D. ryukyuensis*, no organized testes, vitelline glands, nor copulatory organs were observed in the groups fed with tapeworms, suggesting that cocoon production was unlikely to take place. The compounds in tapeworms may differ from those required for the full development of organized sexual structures, although they may induce certain changes at the gene expression and cellular levels. Sex-inducing substances may be specific to the flatworm sub-group, conserved among planarians, monogeneans, and flukes. However, monogeneans and flukes constitute nearly 77% of parasitic flatworms,^1^ causing tremendous damage (approximately 8 billion US$/y worldwide)^1^ related to human health and livestock-fish farming. Thus, the scope of application of our findings can be expanded to numerous parasitic flatworms, providing a rational basis for researchers to apply knowledge of the reproductive biology of one flatworm species to others.

In a previous study on the land planarian *B. nobile*,^22^ full sex-inducing effects were observed in the HPLC fraction combining the tryptophan peak with a subsequently eluted fraction. However, full sex-inducing effects eventually disappeared after further fractionation, and only some fractions caused ovarian induction. This suggests two possibilities: (1) sex-inducing substances are composed of multiple ovary-inducing substances that work in an additive and synergistic manner; or (2) there might be a single, crucial sex-inducing substance; however, the amounts in the fraction currently examined are too low (e.g., because of peak broadening resulting in lower purification and detection efficiencies), which only induced ovaries. The latter possibility is more likely, as Kitamura et al.^22^ used ethyl acetate in the first extraction step to separate the sex-inducing substances from the large amounts of tryptophan, which probably also reduced the amount of sex-inducing substances. Moreover, *B. nobile* had to be collected from the field during the breeding season, and the sex-inducing effects were unstable, varying from batch to batch, probably because of differences in field conditions. In the present study, we established a new method, wherein sex-inducing substances were efficiently secured by first extracting them with water (different from Kitamura et al.^22^). Thereafter, the large amount of tryptophan extracted together was separated via open-column chromatography. Combined with the usage of the fluke *C. calicophorum*, we consider that this resulted in Fr. M30 having full sex-inducing effects strong enough to ensure that the activity was not lost on subsequent fractionation via reverse-phase HPLC. However, no consistent peaks were observed in the effective HPLC fraction, and quantities still need to be secured for isolation and structure estimation. Conversely, this also means that the sex-inducing substance(s) is effective even at very low concentrations. The concentrations used in feeding bioassays were determined based on the concentration of tryptophan (see “STAR Methods”), which was abundant in the fraction with full sex-inducing effects,^27^ but no single effective substance was found even in that range. This indicates that the sex-inducing substance may be an unknown substance that cannot be detected in the present metabolome analysis. However, the possibility (1) cannot be excluded at this stage unless the sex-inducing substance is identified. In our study, we identified 18 ovary-inducing substances. A key future direction is verifying whether the sex-inducing effect is increased when several substances are combined, which was not possible in the current study. Although the feeding bioassay is simple and convenient, one of its weaknesses is the difficulty of verifying the additive and synergistic effects of multiple substances as planarians are reluctant to eat when multiple substances are added to their regular food (chicken liver homogenate), probably because of a shift in the balance of taste. In the future, careful feeding bioassays, which consider the relative balance of each ovary-inducing substance in the body, would be required to verify the synergistic effects of combining substances.

Six out of the 18 ovary-inducing substances were associated with purine metabolism. When combined with the results of the transcriptome analysis, purine metabolism undoubtedly plays a very important role, and three potential working mechanisms can be considered at this stage. First, the metabolites may have worked indirectly because of a structure-activity relationship (SAR) with crucial substances responsible for the full sex-inducing effects. Second, the metabolites may have affected planarian physiology by altering the balance of purine metabolism toward that in the sexual state, leading to the production of crucial substances, although in insufficient amounts. In both cases, such crucial substances may be unknown purine metabolites (e.g., alkaloids) specific to the flatworm sub-group, which could not be identified by the current metabolome database used in the present study. For example, xanthosine is an important precursor of purine alkaloids, such as caffeine and theobromine,^38^ which are structurally similar to adenosine as a messenger molecule and act as antagonists. Third, such metabolites may be directly involved in the signaling pathways for sexual maturation because xanthosine, GTP, and GDP are associated with numerous biological processes. For example, a recent study reported that *in vitro* xanthosine treatment increased mammary stem cell populations by enhancing symmetric cell division,^39^ although the molecular mechanism of action remains unclear. GTP and GDP are important molecules that bind to G-proteins and switch their active and inactive states which affects G-protein coupled receptor (GPCR) function. Interacting with diverse types of ligands, GPCRs transmit signals from the external environment of the cell through downstream signaling cascades and play essential roles in numerous biological processes, such as growth, differentiation, and reproduction.

Consistent with the lack of full sex-inducing effects, lower purine metabolism in tapeworms was inferred from a study comparing ribonucleotide levels among several parasites using HPLC analysis. Adenine (AMP+ADP+ATP) and guanine (GMP+GDP+GTP) nucleotide levels in the fluke *F. hepatica* were 3.07 ± 0.50 and 0.68 ± 0.18 (μmol/g fresh weight ±SD), respectively, while those in the tapeworm *Moniezia expansa* were 1.72 ± 0.41 and 0.34 ± 0.10, respectively.^40^ The importance of purine metabolism in parasitic flatworm sexual maturation was indicated in the fluke *Schistosoma japonicum*.^41^ The growth, development, and reproduction of schistosomes are known to be retarded in immunodeficient mammalian hosts, resulting in decreased egg-laying.^42^^,^^43^^,^^44^ Liu et al. compared the metabolic profiles of *S. japonicum* worms collected from severe combined immunodeficient (SCID) mice and normal control (BALB/c) mice, highlighting purine metabolism as one of the enriched sets of the differential metabolites in both male and female worms.^41^ Future studies to clarify the sex-inducing substances and the roles of purine metabolism in planarians, with a focus on gene regulation associated with bioactive compounds, for example, would contribute to a deeper and more comprehensive understanding of the sexual maturation of parasitic flatworms.

In previous studies, d-Trp, l-Trp, and serotonin were identified as ovary-inducing substances.^24^^,^^27^ Together with 5-hydroxytryptophan identified in the present study, a total of four ovary-inducing substances have been identified from tryptophan metabolism. The direct relationship between purine and tryptophan metabolism in the sexualization of planarians is not yet known. A previous transcriptome analysis^24^ comparing asexual and sexual worms yielded 10,059 contigs identified as sexual DEGs (at a fold-change cutoff of >2), with differences in amino acid metabolism, particularly tryptophan, being the main focus, as tryptophan has already been identified as an ovary-inducing substance.^27^ In fact, however, purine metabolism was also identified as one of the metabolic pathways highly enriched in sexual compared to asexual worms, although not paid much attention. The present study used a cocoon transcriptome with the keyword “vitellocyte,” further narrowed down purine metabolism as the sole metabolic pathway, indicating its importance for considering sex-inducing substances. Given our HPLC data (Figure 5B) and a previous study,^22^ which suggest the presence of a crucial sex-inducing substance previously hidden behind the overwhelmingly large amount of tryptophan, it may be an unknown purine metabolite unique to the sub-group of flatworms. Because the feeding bioassays in our study identified various metabolites as ovary-inducing substances, different compounds seem to work in concert for sexualization, with tryptophan metabolites representing such enhancers. At present, two possible relationships between purine and tryptophan metabolism can be considered; (i) the crucial sex-inducing substance, which happens to be in the vicinity of tryptophan retention time, may be a purine metabolite, such as a purine alkaloid; (ii) purine metabolites also act as enhancers, functioning in concert with tryptophan metabolites. Intriguingly, there are some cases of alterations in tryptophan and purine metabolism in patients with psychiatric disorders.^45^^,^^46^ The body structure of asexual planarians is relatively simple, with the main organs belonging to the feeding system (e.g., the pharynx and intestine) and the nervous system, including the brain. The function of the nervous system seems to be important in the sexualization process, as in the planarian *S. mediterranea*, neuropeptide NPY8 and its GPCR were reported as key regulators of sexual maturation.^47^^,^^48^ The tryptophan metabolites, including serotonin as an ovary-inducing substance,^24^ and the purine metabolites identified in the present study may also have such roles within the nervous system during flatworm sexual maturation.

### Limitations of the study

The substances capable of sexualizing planarians were present in parasitic flatworms. Sex-inducing substances in the planarian *B. nobile* and the parasitic flatworm *C. calicophorum* were likely the same compound(s) eluting around the retention time of tryptophan, previously remaining hidden by the high peak of tryptophan. However, without NMR or other structural estimation following purification, it cannot be concluded that they are the same substance conserved among planarians and parasitic flatworms. The compounds that can be identified by transcriptome and metabolome analyses are limited to those that are known and annotated in databases. Unknown compounds cannot be recognized using the above approach. Further purification based on our methods combining open-column chromatography and reverse-phase HPLC should enable the isolation and identification of unknown, uniquely evolved flatworm sub-group-specific sex-inducing substance(s), as suggested by our study.

Further studies are also needed to verify whether parasitic flatworms indeed utilize sex-inducing substances for their own sexual maturation. Just as the addition of vitamin C has only recently been shown to improve the culture medium for the fluke *S. mansoni*,^49^ the 18 ovary-inducing substances identified in our study, which include vitamin C, may improve the culture medium for the *in vitro* sexual maturation of parasitic flatworms. Future studies on how these ovary-inducing substances and HPLC fractions isolated in the present study affect the *in vitro* sexual maturation will clarify the role of sex-inducing substances in parasitic flatworms.

The future identification of sex-inducing substances would corroborate similarities in the regulation of reproductive mode among flatworms, in addition to the previously reported similarities in stem cells between the planarians and parasitic flatworms.^2^^,^^17^^,^^18^^,^^19^^,^^20^ This suggests the presence of a common molecular mechanism that regulates stem cell behavior via sex-inducing substances to initiate sexual development. Our study provides a platform for understanding and controlling the life cycles of parasitic flatworms for future parasitology studies and applications, for instance, anti-parasitic drug development based on novel purine alkaloids as physiological modulators.

## STAR★Methods

### Key resources table

**Table:** 

REAGENT or RESOURCE	SOURCE	IDENTIFIER
**Antibodies**		

alkaline phosphatase-conjugated anti-DIG antibodies	Roche	Cat#11093274910; RRID AB_514497

**Chemicals, peptides, and recombinant proteins**		

11-Aminoundecanoic acid	Ark Pharm (USA)	Cat#AK111174
2-Phenylethylamine hydrochroride	Sigma-Aldrich (USA)	Cat#P6513-25G
DL-3-Hydroxybutyric Acid	Tokyo Chemical Industry (Japan)	Cat#H0228
3-METHOXYTYRAMINE HYDROCHLORIDE	MP Biomedicals (USA)	Cat#105665
3-O-Methyl-L-DOPA Monohydrate	Toront Reserarch Chemicals (Canada)	Cat#M303815
2′-Deoxyadenosine 3':5′-cyclic monophosphate sodium salt	Santa Cruz Biotechnology (USA)	Cat#sc-214051
4-Acetamidobutyric acid	Santa Cruz Biotechnology (USA)	Cat#sc-276980
4-Pyridoxic acid	Sigma-Aldrich (USA)	Cat#P9630-25MG
5-Hydroxy-L-Tryptophan	Sigma-Aldrich (USA)	Cat#107751-1G
L(+)-Ascorbic Acid	Nacalai tesque (Japan)	Cat#03420-52
L-DOPA	Nacalai tesque (Japan)	Cat#14211-81
Dopamine Hydrochloride	Nacalai tesque (Japan)	Cat#14212-71
Thymidine 5′-diphosphate sodium salt	Cosmo Bio (Japan)	Cat#sc-215980
2-Aminoethanol	Wako (Japan)	Cat#016-12453
Guanosine 5′-diphosphate sodium salt	Sigma-Aldrich (USA)	Cat#G7127-25MG
D-Glucuronic acid	Sigma-Aldrich (USA)	Cat#G5269-10G
Guanosine 5′-triphosphate sodium salt	Sigma-Aldrich (USA)	Cat#G8877-25MG
Heptanoic Acid	Nacalai tesque (Japan)	Cat#14316-92
Histamine (free base)	Nacalai tesque (Japan)	Cat#18111-71
Homovanillic acid Fluorimetric reagent	Sigma-Aldrich (USA)	Cat#H1252-100MG
Hypoxanthine	Nacalai tesque (Japan)	Cat#17487-01
L-Kynurenine	Sigma-Aldrich (USA)	Cat#K8625-25MG
L-Homoserine	Sigma-Aldrich (USA)	Cat#H1030
N-Me-Glu-OH	bachem (Switzerland)	Cat#4002660.0001
(S)-1-Methylpyrrolidine-2-carboxylic acid hydrate	Ark Pharm (USA)	Cat#AK-46644
NADP, disodium salt, approx. 98%	Roche (Switzerland)	Cat#10128031001
O-Phospho-L-serine	Nacalai tesque (Japan)	Cat#27833-41
(±)-Octopamine hydrochloride	Sigma-Aldrich (USA)	Cat#O0250-1G
Sodium pyrophosphate tetrabasic decahydrate	Sigma-Aldrich (USA)	Cat#S6422-100G
(±)-Mevalonolactone	Sigma-Aldrich (USA)	Cat#M4667-1G
Ser-Glu	AnaSpec Inc	Cat#AS-65126-SE
Terephthalic acid	Sigma-Aldrich (USA)	Cat#185361-5G
Tyr-Glu	AnaSpec Inc	Cat#65126-YE
Tyramine	MP Biomedicals (USA)	Cat#A0302
Xanthosine dihydrate	Sigma-Aldrich (USA)	Cat#X0750-5G
L-Tyrosine	Nacalai tesque (Japan)	Cat#35709-44
6-Phospho-D-Gluconate, trisodium salt	Wako (Japan)	Cat#45190000

**Critical commercial assays**		

Sepasol RNA I Super G	Nacalai tesque (Japan)	Cat#09379-55
TruSeq Stranded mRNA Library Prep for NeoPrep	Illumina (USA)	Cat#NP-202-1001
HiSeq Rapid SBS kit V2	Illumina (USA)	Cat#FC-402-4023

**Deposited data**		

Raw Illumina sequences	This paper	DDBJ: DRA011805

**Experimental models: Organisms/strains**		

*Dugesia ryukyuensis*: OH strain	Hirosaki University	N/A

**Oligonucleotides**		

Primer for DIG-probe synthesis: *TR34905|c0_g1_i1* Forward: ATGGCCTCCGCTGATAAAG	This paper	N/A
Primer for DIG-probe synthesis: *TR34905|c0_g1_i1* Reverse: GCATCCATTCGAAATGACCT	This paper	N/A
Primer for qRT-PCR: *TR34905|c0_g1_i1* Forward: TTTAGAGCAGGGCATGTTCG	This paper	N/A
Primer for qRT-PCR: *TR34905|c0_g1_i1* Reverse: TCGTCCACAACGTCCAATTC	This paper	N/A
Primer for qRT-PCR: *DrY1* Forward: TATGCCTCCACCTCCTCAAG	Nakagawa et al.^12^	N/A
Primer for qRT-PCR: *DrY1* Reverse: CGCCACGATAACCCATAATC	Nakagawa et al.^12^	N/A
Primer for qRT-PCR: *Dryg* Forward: AAATCTATCGTTGCCCGATG	Hase et al.^16^	N/A
Primer for qRT-PCR: *Dryg* Reverse: TCGCATCGTTTTGATGTTTG	Hase et al.^16^	N/A
Primer for qRT-PCR: *Dref1a* Forward: TTGGTTATCAACCCGATGGTG	Sekii et al.^24^	N/A
Primer for qRT-PCR: *Dref1a* Reverse: TCCCATCCCTTGTACCATGAC	Sekii et al.^24^	N/A

**Software and algorithms**		

R	R Development Core Team^50^	https://www.r-project.org/
cutadapt (v1.8.1)	Martin^51^	https://cutadapt.readthedocs.io/en/stable/
Trinity (v2.0.6)	Grabherr et al.^52^	https://github.com/trinityrnaseq/trinityrnaseq/releases
Trinotate (v3.1.1)	Bryant et al.^53^	https://github.com/Trinotate/Trinotate/releases
For HPLC: a software ChromNAV (version 2)	JASCO (Japan)	N/A

**Other**		

Sep-Pak® Light tC_18_ Cartridge	Waters (USA)	Cat#WAT036805
COSMOSIL 75 C18–OPN	Nacalai tesque (Japan)	Cat#37842-95
For HPLC: Develosil C30-UG-5 (4.6 φ × 250 mm)	Nomura Chemical (Japan)	Cat#UG17546250W
For HPLC: Develosil C30-UG-5 (20 φ × 250 mm)	Nomura Chemical (Japan)	Cat#UG175P2250W
For HPLC: PU-2089 Plus Quaternary Gradient Pump	JASCO (Japan)	N/A
For HPLC: UV-2075 Plus Intelligent UV detector	JASCO (Japan)	N/A
For HPLC: LC-Net II/ADC data collector	JASCO (Japan)	N/A

### Resource availability

#### Lead contact

Further information and requests for resources and reagents should be directed to and will be fulfilled by the lead contact, Kiyono Sekii (kiyono.sekii@gmail.com).

#### Materials availability

This study did not generate new unique reagents.

### Experimental model and subject details

#### Planarians

An exclusively asexual strain (OH) of the planarian *D. ryukyuensis* was established by Dr. S. Ishida at Hirosaki University (Hirosaki, Japan). The OH strain was maintained at 20°C in autoclaved tap water and fed organic chicken liver (Champool, Kanagawa, Japan). Under these conditions, the OH strain is exclusively fissiparous and never reproduces sexually, resulting in the establishment of a clonal asexual population.

The planarian *B. brunnea* was collected at small rivers in Aomori prefecture, Japan, in the springs of 2015–2017.

#### Monogeneans

The monogenean *N. hirame*, infecting the buccal cavity of farmed *Paralichthys olivaceus*, was collected using forceps in Wakayama prefecture, Japan, in May 2017. The monogenean *N. girellae* was obtained in Wakayama prefecture, Japan, in 2018, by exposing infected farmed *Seriola dumerili* to freshwater and collecting dislodged worms. No fish were killed during parasite collection.

#### Flukes

The fluke Hybrid *Fasciola* strain wuh15-2 used in this study was originally isolated in 2007 from a cow in Wuhan, China, and has been maintained in Wistar rats and *Lymnaea ollula* at the Laboratory of Veterinary Parasitology, Faculty of Agriculture, Iwate University, Japan. The fluke *C. calicophorum* was collected in 2018 from cattle slaughtered at a meat sanitation test facility located in Shiwa, Japan. The fluke *S. mansoni* (Puerto Rican strain) was maintained in the laboratory using ICR mice and the snail *Biomphalaria glabrata* as an intermediate host. At 7 weeks post-infection, adult worms were recovered from the infected mice. The collected adult worms were divided into males and females based on microscopic observation.

#### Tapeworms

The tapeworm *H. microstoma* had been maintained as a laboratory strain using *Tribolium confusum* and mice at Asahikawa Medical University. The adult worms were recovered from the bile ducts or small intestines of experimentally infected mice. The tapeworm *M. benedeni* was collected from cattle slaughtered at livestock farms located in Gifu prefecture, Japan, in 2018. The larval-stage parasites of the tapeworm *D. hottai* were prepared from *Hypomesus japonicus* in Hokkaido, Japan. Recovered plerocercoids were administered orally to Wistar rats and maintained at the Laboratory of Veterinary Parasitology, Faculty of Agriculture, Iwate University. The tapeworm *S. erinaceieuropaei* was collected from house cats at animal hospitals in Japan between 2017 and 2018.

### Method details

#### Preparation of Fr. M0, M10, and M100 from various flatworms using Sep-Pak® Light tC_18_ Cartridge

Fractionation for the bioassays was conducted as follows^15^ (Figure S1). Approximately 4 g wet weight of various sexually mature flatworms (Figure 1) were used. Each flatworm was homogenized in 240 mL of PBS (34 mM NaCl, 0.68 mM KCl, 2.5 mM Na_2_HPO_4_, and 0.45 mM KH_2_PO_4_; pH 7.4). The homogenate was centrifuged at 16,000 × *g* for 30 min at 4°C. The supernatant was filtered using a 0.22 μm Millex®GV filter (Millipore, Carrigtwohill, Cork, Ireland) and then centrifuged at 120,000 × *g* for 30 min at 4°C. It was subsequently freeze-dried and dissolved in water, yielding 36 mL of extract. The extract was further loaded onto a Sep-Pak® Light tC_18_ Cartridge (Waters, Milford, MA, USA), whose silica-based bonded phase with strong hydrophobicity absorbed the substances in the extract. The substances were sequentially eluted using 0, 10, and 100% aqueous methanol to create 96 mL of Fr. M0, Fr. M10, and Fr. M100, respectively. The obtained fractions were freeze-dried, and the resulting powders were used for feeding bioassays.

#### Fractionation of sex-inducing substances from *B. nobile* and *C. calicophorum* on open-column chromatography

A new fractionation method for sex-inducing substances was conducted as follows (Figure S5). Approximately 8 g wet weights of *B. nobile* and *C. calicophorum* were used. Each flatworm was homogenized in 480 mL of PBS (34 mM NaCl, 0.68 mM KCl, 2.5 mM Na_2_HPO_4_, and 0.45 mM KH_2_PO_4_; pH 7.4). The homogenate was centrifuged at 16,000 × *g* for 30min at 4°C. The supernatant was filtered using a 0.22 μm Millex®GV filter (Millipore, Carrigtwohill, Cork, Ireland) and then centrifuged at 120,000 × *g* for 30min at 4°C. It was subsequently freeze-dried and dissolved in water, yielding 100 mL of extract. The extract was further separated via open-column chromatography, using 50 g of ODS (COSMOSIL 75 C18–OPN, Nacalai Tesque, Kyoto, Japan) and an Econo-Column (50 φ × 500 mm, Bio-Rad, CA, USA). The substances absorbed in ODS were sequentially eluted using 0%, 10%, 30%, 50%, 70%, and 100% aqueous methanol to create 300 mL of Fr. M0, Fr. M10, Fr. M30, Fr. M50, Fr. M70, and Fr. M100, respectively. The obtained fractions were freeze-dried, and the resulting powders were used for feeding bioassays and HPLC analysis.

#### Reverse-phase HPLC

The powder of Frs. M10 and M30 obtained via a new fractionation method using open-column chromatography was dissolved in 8 mL of water and further subjected to reverse-phase HPLC for analytical and fractionation purposes.

Reverse-phase HPLC was performed using a column C30-UG-5 (4.6 φ × 250 mm for analytical purposes and 20 φ × 250 mm for fractionation purposes, Nomura Chemical, Aichi, Japan) and an HPLC system (JASCO PU-2089 Plus Quaternary Gradient Pump, JASCO UV-2075 Plus Intelligent UV detector, JASCO LC-Net II/ADC data collector, and a software ChromNAV version 2, JASCO, Tokyo, Japan). The development started with 15% aqueous acetonitrile (MeCN), followed by washing with MeCN (HPLC-grade, Nacalai Tesque, Japan). The flow rate was set to 5.0 mL/min. The detection wavelength was at 290 nm.

For analyzing the quantities of Trp contained in Frs. M10 and M30, 16 μL out of 8 mL of each fraction (i.e., derived from 0.016 g of flatworm) were used. For fractionating sex-inducing substances, 2 mL of Fr. M30 (i.e., derived from 2 g of flatworm) were injected into the HPLC system over four times of injection. HPLC fractions corresponding to the peaks of interest were manually collected by visually checking the peak patterns identified on the PC monitor. The obtained fractions were freeze-dried and used for feeding bioassays.

#### Feeding bioassay

Feeding bioassays were conducted to investigate the sex-inducing effects of (i) various purified flatworm fractions and (ii) metabolites of interest (Tables S1 and S2) by feeding the asexual *D. ryukyuensis* OH strain worms with test food. For (i), the test food was prepared by mixing freeze-dried powders of aqueous methanol fractions or HPLC fractions with chicken liver homogenates. Ten microliters of chicken liver homogenate was used per one asexual worm (e.g., 300 μL for the assay of 30 worms). The control worms were fed chicken liver homogenates. The test food was freeze-dried, cut into 28 pieces, and fed to the worms every day for 28 d (4 weeks) in a 40 mm plastic Petri dish. After feeding, the worms were kept at a density of 5 worms per 90 mm plastic Petri dish filled with fresh autoclaved tap water. At this density, fission of asexual worms was rarely observed during the 4 weeks of feeding.

For (ii), the test food was prepared by mixing various concentrations of each metabolite of interest (15.5–15,500 ng/worm/d, see “Dataset S5” for more details) with chicken liver homogenates, and feeding bioassays were performed as described above. The metabolite concentrations to mix were determined as follows. First, a reference concentration was determined based on previous findings on tryptophan. Tryptophan has been reported as an ovary-inducing substance in *D. ryukyuensis* and was estimated to be present in 2,160 μg in Fr. M0+M10 derived from 4 grams of *B. brunnea* worms (wet weight).^27^Since reverse-phase HPLC analysis showed no peaks more prominent than that of tryptophan in the fraction with sex-inducing activity, we assumed that a substance with sex-inducing activity is unlikely to be present at quantities greater than of tryptophan. Therefore, based on the feeding bioassay examining the effect of tryptophan in Kobayashi et al.,^27^ a reference “concentration 1” in the present study was set to 1,550 ng/worm/d. We considered that a few points of 10-fold increase or decrease (0.01–10 times) in concentration would cover the effective concentration if the metabolite of interest is a sex-inducing substance.

After 4 weeks of feeding, we examined all worms under an Olympus SZX10 microscope (Olympus Corporation, Tokyo, Japan). The presence of externally observable ovaries, copulatory organs, and genital pores was scored, and the stages were classified: worms that did not show morphological changes were classified as stage 0; those that initiated ovary development as stage 1–2 (before the point of no-return); those with developing ovaries and copulatory organs as stage 3–4 (after the point of no-return); those that developed ovaries, copulatory organs, and genital pores as stage 5–6 (after the point of no-return). Two worms that exhibited the most developed reproductive organs within each group were selected for imaging (Olympus DP22 digital camera, Olympus Corporation) and then embedded for histological sectioning. Further, the 10 or 11 most sexually developed worms from each feeding bioassay group were selected for qRT-PCR analysis to examine the development of the ovary, testis, and vitellaria.

#### qRT-PCR

Total RNA was extracted from individual worms using Sepasol RNA I Super G (Nacalai Tesque, Kyoto, Japan) following the manufacturer’s instructions and treated with TURBO™ DNase using the TURBO DNA-free kit (Thermo Fisher Scientific, Waltham, MA, USA). Next, 0.5 mg of total RNA was used to prepare cDNA using the ReverTra Ace kit (Toyobo, Tokyo, Japan). qRT-PCR was performed using the KAPA SYBR Fast qPCR master mix kit (KAPA Biosystems, Wilmington, MA, USA) and a DNA Engine Opticon 2 system (Bio-Rad Laboratories, Hercules, CA, USA) following the manufacturers’ instructions.

The following primers were used: ovary marker gene *TR34905|c0_g1_i1*, forward 5′-TTTAGAGCAGGGCATGTTCG-3′ and reverse 5′-TCGTCCACAACGTCCAATTC-3′; testis marker gene *DrY1*,^12^^,^^23^^,^^24^ forward 5′-TATGCCTCCACCTCCTCAAG-3′ and reverse 5′-CGCCACGATAACCCATAATC-3′; vitellaria marker gene *Dryg*,^16^ forward 5′-AAATCTATCGTTGCCCGATG-3′ and reverse 5′-TCGCATCGTTTTGATGTTTG-3′. The *D. ryukyuensis* homolog of the gene encoding elongation factor 1 alpha, *Dref1a*, was used as the internal control^23^^,^^24^ (forward 5′-TTGGTTATCAACCCGATGGTG-3′ and reverse 5′-TCCCATCCCTTGTACCATGAC-3′). The cycling conditions were as follows: 1min at 95°C; 40 cycles of 2s at 95°C and 30s at 60°C; 1min at 65°C. Differences in the obtained threshold cycle (Ct) values between samples were calculated using the ΔΔCt method. The following formulas were used for ΔCt (where ΔCt = Ct [target gene] – Ct [internal control]); ΔΔCt (where ΔΔCt = ΔCt [sample] – the average of ΔCt [calibrator]). The calibrators were the control group in the feeding bioassay or asexual worms when investigating *TR34905|c0_g1_i1* expression. Relative expression was calculated as 2^−ΔΔCt^.

#### Histology

After feeding bioassays, the worms that exhibited the most developed reproductive organs within each group were selected and embedded for histological sectioning. The worms were relaxed in cold 2% (v/v) HCl in 5/8 Holtfreter’s solution^54^ for 5 min and were then fixed in 4% paraformaldehyde and 5% methanol in 5/8 Holtfreter’s solution for 3h at room temperature (RT). The fixed specimens were dehydrated through an ethanol series, cleared in xylene, and embedded in the Paraplast Plus embedding medium (Sigma-Aldrich Co., St. Louis, MO, USA). Embedded specimens were cut into 5 μm thick sections using a microtome (Leica RM2125 RTS, Leica Microsystems Ltd., Wetzlar, Germany). Tissue sections were placed on slide glasses coated with 5 mg/mL egg albumin (Nacalai Tesque, Japan) in 50% glycerin (FUJIFILM Wako Pure Chemical Corporation, Osaka, Japan), stretched at 40°C overnight, and stained with Mayer’s Hematoxylin (No. 3000-2, Muto Pure Chemicals, Tokyo, Japan) and 1% Eosin Y solution (No. 3200-2, Muto Pure Chemicals, Japan).

#### Metabolome analysis

Metabolome analysis was conducted by Human Metabolome Technologies, Inc. (HMT, Tsuruoka, Japan)^55^^,^^56^^,^^57^ using a Basic Scan package. Briefly, 5 asexual *D. ryukyuensis* worms (30 mg) were collected as an asexual sample, 3 sexual *D. ryukyuensis* worms (36 mg) as a sexual sample, and 10 freshly laid *D. ryukyuensis* cocoons (collected within 24 h of oviposition) (30 mg) as a cocoon sample. All samples were frozen at −80°C and transported to HMT on dry ice. The asexual and sexual worms were starved for 2 weeks prior to sampling. Each sample was added into 1,500 μL of 50% acetonitrile aqueous solution (v/v) containing internal standards (HMT). The samples were homogenized using a BMS-M10N21 homogenizer (BMS, Tokyo, Japan) and centrifuged at 2,300 × *g* for 5 min at 4°C. Next, 800 μL of the upper aqueous layer (400 μL for each of the anion and cation modes) was centrifugally filtered through a 5 kDa cutoff filter (ultrafree MC PLHCC, HMT) at 9,100 × *g* for 120 min at 4°C. The filtrate was resuspended in 50 μL of water. Metabolites were measured in the cationic and anionic modes of capillary electrophoresis time-of-flight mass spectrometry (Agilent Technologies, Waldbronn, Germany). Peak information, including mass-to-charge ratio (m/z), migration time (MT), and peak area, was obtained using the automatic integration software MasterHands ver. 2.16.0.15 (Keio University, Tokyo, Japan). The peaks with a signal-to-noise ratio higher ≥3 were aligned according to the m/z value and normalized MT. Target metabolites were assigned using the HMT standard library and the known-unknown peak library based on m/z and MT. The tolerance was ±0.5 min in MT and ±10 ppm in m/z.

#### RNA-seq

Freshly laid *D. ryukyuensis* and *B. brunnea* cocoons (collected within 24 h of oviposition) were used for RNA sequencing. Three biological replicates were used for each species. Total RNA was extracted from each cocoon using Sepasol RNA I Super G (Nacalai Tesque, Kyoto, Japan) following the manufacturer’s instructions. Total RNA was treated with TURBO™ DNase using the TURBO DNA-free kit (Thermo Fisher Scientific) and was purified using the RNeasy MicroKit (QIAGEN, Hilden, Germany) following the manufacturers’ instructions. After removing residual DNA via on-column DNase digestion using the RNeasy Mini Kit (QIAGEN), RNA integrity was validated using an Agilent 2100 BioAnalyzer (Agilent Technologies, Santa Clara, CA, USA). The cDNA libraries were prepared using the TruSeq Stranded mRNA Library Prep for NeoPrep (Illumina, San Diego, CA, USA) on a fully automated library preparation instrument, NeoPrep Library Prep System (Illumina, discontinued) following the manufacturer’s instructions, with 120 ng of total RNA as starting material. The enriched cDNA libraries were validated using an Agilent 2100 BioAnalyzer (Agilent Technologies), and the concentration of libraries was quantified using a Qubit HS RNA kit (Thermo Fisher Scientific) and digital droplet PCR (Bio-Rad, Hercules, CA, USA). Multiplex sequencing of paired-end reads was performed using an equimolar mixture of the final cDNA libraries and HiSeq Rapid SBS kit V2 (Illumina) on an Illumina HiSeq2500 system (Illumina).

#### Raw data processing and *de novo* assembly

The raw Illumina reads were cleaned up using cutadapt (v1.8.1).^51^ Low-quality ends (quality-value [QV] < 30) and adapter sequences were trimmed. To build a comprehensive set of reference transcript sequences, cleaned reads derived from all libraries (asexual, experimentally induced sexual, and innately sexual planarians) were pooled and input into the Trinity (v2.0.6)^52^*de novo*RNA-seq assembler in the paired-end mode using default parameters.

#### Functional annotation of the cocoon transcriptome

Annotation of the *de novo* assembled transcriptome of freshly laid *D. ryukyuensis* and *B. brunnea* cocoons was performed using the software Trinotate (v3.1.1)^53^ with default settings.

#### Isolation of sets 1 and 2 genes

Using information regarding the DEGs from our previous study,^24^ we obtained the sexual DEGs of the planarian *D. ryukyuensis* with criteria of false discovery rate (FDR) < 0.05 and log_2_ fold-change of sexual/asexual >0. Using these sexual DEGs, gene Sets 1 and 2 were isolated as follows (also see Figure S6). First, using the sexual DEGs as query sequences, a Basic Local Alignment Search Tool (BLAST) search was performed against the *de novo* assembly transcript models of the planarian *D. ryukyuensis* cocoons (Dataset S1). Sexual DEGs with similarity to the transcripts in *D. ryukyuensis* cocoons (with an e-value cutoff of e-120) were selected and listed as Set 1 genes (Dataset S2). Namely, the Set 1 genes are a subset of the sexual DEGs, which are also expressed in freshly laid cocoons, in addition to being more highly expressed in sexual than asexual worms. Next, using the Set 1 genes as query sequences, we performed a BLAST search against the *de novo* assembly transcript models of the planarian *B. brunnea* cocoons (Dataset S3). Set 1 genes with similarity to the transcripts in *B. brunnea* cocoons (with an e-value cutoff of e-30) were selected and listed as Set 2 genes (Dataset S4). Namely, Set 2 genes are a subset of Set 1 genes, conserved between the planarians *D. ryukyuensis* and *B. brunnea*.

#### KEGG pathway enrichment analysis of sets 1 and 2 genes

First, the UniProt ID (https://www.uniprot.org) of Sets 1 and 2 genes were obtained using already available information from KEGG annotation presented in our previous study^24^ and the ID converting list (Dataset S6). The ID converting list was prepared using the KEGG API (https://www.kegg.jp/kegg/rest/keggapi.html). Using the obtained UniProt ID and Metascape software (https://metascape.org/gp/index.html),^58^ KEGG pathway enrichment analysis of gene Sets 1 and 2 was performed.

#### Isolation of the ovary marker gene

The expression pattern of *TR34905|c0_g1_i1* was confirmed quantitatively via qRT-PCR and qualitatively using whole-mount*in situ* hybridization. The primers used for qRT-PCR are described in the “qRT-PCR” section. Primers used to synthesize whole-mount*in situ* hybridization probes were forward, 5′-ATGGCCTCCGCTGATAAAG-3′ and reverse, 5′-GCATCCATTCGAAATGACCT-3′. Digoxigenin (DIG)-labeled anti-sense RNA probes were synthesized *in vitro* using DIG-11-UTP (Roche, Mannheim, Germany) and the MEGA script T7 kit (Thermo Fisher Scientific). Whole-mount*in situ* hybridization was performed as follows.^59^ Worms were relaxed in cold 2% (vol/vol) HCl in 5/8 Holtfreter’s solution^54^ for 5 min and fixed in 4% paraformaldehyde and 5% methanol in 5/8 Holtfreter’s solution at 4°C for 3 h. The fixed specimens were bleached in hydrogen peroxide/methanol [1:4 (vol/vol)] under fluorescent light at RT for approximately 12 h. After hydration in a series of decreasing methanol concentrations, the specimens were treated with proteinase K (20 μg/mL; Nacalai Tesque, Kyoto, Japan) in PBTw (0.1% Tween 20 in phosphate-buffered saline) at 37°C for 10 min for asexual worms or 15 min for sexual worms and post-fixed in 4% PFA solution at RT for 30 min. The specimens were then washed three times in PBTw for 10 min. They were then incubated in a prehybridization solution [50% formamide, 5 × SSC, 100 μg/mL yeast tRNA (Roche), 100 μg/mL heparin, and 0.1% Tween 20] at 56°C for 1 h and hybridized with a DIG-labeled antisense RNA probe (approximately 50 ng/mL) in prehybridization solution supplemented with 10% dextran sulfate at 56°C for 16 h. After hybridization, the specimens were washed six times in a solution of 50% formamide, 5 × SSC, and 0.1% Tween 20 at 56°C for 30 min; once in a solution of 20% formamide, 2 × SSC, and 0.1% Tween 20 at 56°C for 30 min; and two times in a solution of 2% formamide, 0.2 × SSC, and 0.1% Tween 20 at 56°C for 30 min. Then, they were washed three times in MABTw (pH 7.5, 100 mM maleic acid, 150 mM NaCl, 0.1% Tween 20) at RT for 10 min. They were incubated in a blocking solution [MABTw containing 1% blocking reagent (Roche) and 10% sheep serum] at RT for 1 h and then incubated in the blocking solution with alkaline phosphatase-conjugated anti-DIG antibodies (1:2,000, Roche, catalog number 11093274910) at RT for 3 h. Then, the specimens were washed in MABTw four times for 15 min and another four times for 30 min and then incubated with TMN solution [100 mM Tris-HCl (pH 9.5), 50 mM MgCl_2_, 100 mM NaCl, and 8.3% polyvinyl alcohol] for 15 min. The color development reactions were developed at 20°C through incubation in TMN solution containing 170 μg/mL of nitro-blue tetrazolium (Roche) and 175 μg/mL of 5-bromo-4-chloro-3′-indolyphosphate (Roche). Specimen examination and imaging were performed using a digital microscopy setup with an Olympus SZX10 microscope (Olympus Corporation) and an Olympus DP22 digital camera (Olympus Corporation). Three biological replicates were used for asexual and sexual worms.

### Quantification and statistical analysis

Statistical tests were performed using R v3.6.3 (R Foundation for Statistical Computing, Vienna, Austria).^50^ Detailed results are available in Dataset S5.

To analyze the feeding bioassays, we used the two-tailed Fisher’s exact test with the number of worms that developed past the point of no-return to examine the sex-inducing effects of the test foods toward the OH strain, which has been exclusively asexual for >40 years. The ovary-inducing effects of the candidate sex-inducing substances were calculated by dividing the number of individuals with developing ovaries by the total number of individuals. The odds ratios were calculated in comparison with d-Trp [ovary-inducing effect of 48.8% (41/84)], which has already been reported as the ovary-inducing substance in a previous study.^27^

For qRT-PCR analysis, statistical tests were performed on the ΔΔCt values. For undetectable gene amplification, which was often the case for the testis marker gene *DrY1* in the asexual control worms, expression was treated as not available (N.A.) in the calculations. To compare gene expression levels among the Fr. M0, M10, and M100-fed groups and the control group, the two-tailed Tukey’s honestly significant difference (HSD) test was used. The comparisons between the worms fed the raw female *S. mansoni* and the control worms were performed using either the two-tailed Student’s *t*-test or two-tailed Welch’s *t*-test, depending on whether the data showed unequal variance. We omitted ANOVA tests because we had already developed hypotheses based on the morphological observations.

## Data Availability

• Illumina sequences generated in the present study are available from the DNA Data Bank of Japan Sequence Read Archive (DRA, http://trace.ddbj.nig.ac.jp/dra/) under the accession numberDDBJ: DRA011805, which is also listed in the key resources table. The data are publicly available as of the date of publication. The *de novo* assemblies of the planarian *D. ryukyuensis* and *B. brunnea* cocoon transcript models are available as Datasets S1 and S3, respectively. The gene annotation lists for these transcript models were not used in the present study but are available as Dataset S7. The lists of contig IDs for Sets 1 and 2 genes are available as Datasets S2 and S4, respectively. The results of feeding bioassays and qRT-PCR, as well as their statistical analyses, are available as Dataset S5. The UniProt converting list used in the present study is available as Dataset S6. Microscopy and HPLC data reported in this paper will be shared by the lead contact upon request. • This paper does not report original code. • Any additional information required to reanalyze the data reported in this paper is available from the lead contact upon request.

## Supplemental information

Document S1. Figures S1–S8 and Tables S1 and S2

Dataset S1. The de novo assembly of the planarian D. ryukyuensis cocoon transcript model, related to STAR Methods

Dataset S2. List of contig IDs for gene Set 1, related to Figure 6

Dataset S3. The de novo assembly of the planarian B. brunnea cocoon transcript model, related to STAR Methods

Dataset S4. List of contig IDs for gene Set 2, related to Figure 6

Dataset S5. Results and statistical analyses of feeding bioassays and qRT-PCR, related to Figures 2, 3, 4, 5, and 7

Dataset S6. UniProt converting list, related to STAR Methods

Dataset S7. Gene annotation lists of the de novo assembled transcript models of the planarian D. ryukyuensis and B. brunnea cocoons, related to STAR Methods
