# Supplementary material for: Sex-inducing effects toward planarians widely present among parasitic flatworms
Source: iScience. 2022 Dec 8;26(1):105776. doi: 10.1016/j.isci.2022.105776 (PMC9804148; doi:10.1016/j.isci.2022.105776)
Supplement: Document S1. Figures S1–S8 and Tables S1 and S2 [file mmc1.pdf]

iScience, Volume *26*

Supplemental information

Sex-inducing effects toward planarians widely present among parasitic flatworms

Kiyono Sekii, Soichiro Miyashita, Kentaro Yamaguchi, Ikuma Saito, Yuria Saito, Sayaka Manta, Masaki Ishikawa, Miyu Narita, Taro Watanabe, Riku Ito, Mizuki Taguchi, Ryohei Furukawa, Aoi Ikeuchi, Kayoko Matsuo, Goro Kurita, Takashi Kumagai, Sho Shirakashi, Kazuo Ogawa, Kimitoshi Sakamoto, Ryo Koyanagi, Noriyuki Satoh, Mizuki Sasaki, Takanobu Maezawa, Madoka Ichikawa-Seki, and Kazuya Kobayashi

Supplemental Figures and Tables

Figure S1. Fractionation procedure of biochemical substances contained in sexually mature flatworms using Sep-Pak® Light tC18 Cartridge; related to STAR Methods and Figures 2 and 3.

Figure S2. Additional feeding bioassay with five fractions from the fluke *Calicophoron calicophorum*; related to Figures 2 and 3.

Figure S3. Isolation of the ovary marker gene *TR34905|c0_g1_i1* of *Dugesia ryukyuensis*; related to Figure 4.

Figure S4. Histological evaluation of the sex-inducing effect of the fractions derived from tapeworms; related to Figure 4.

Figure S5. A new fractionation method for sex-inducing substances using open-column chromatography; related to STAR Methods and Figure 5.

Figure S6. Transcriptome analysis overview; related to STAR Methods and Figure 6.

Figure S7. Metabolome analysis overview; related to STAR Methods and Figures 6 and 7.

Figure S8. Feeding bioassay results for xanthosine, pyrophosphate, GTP, and GDP; related to Figure 7.

Table S1. Metabolites detected in asexual worms, sexual worms, and cocoons; related to Figure 7.

Table S2. List of chemical compounds used for the feeding bioassays; related to STAR Methods and Figure 7.

References

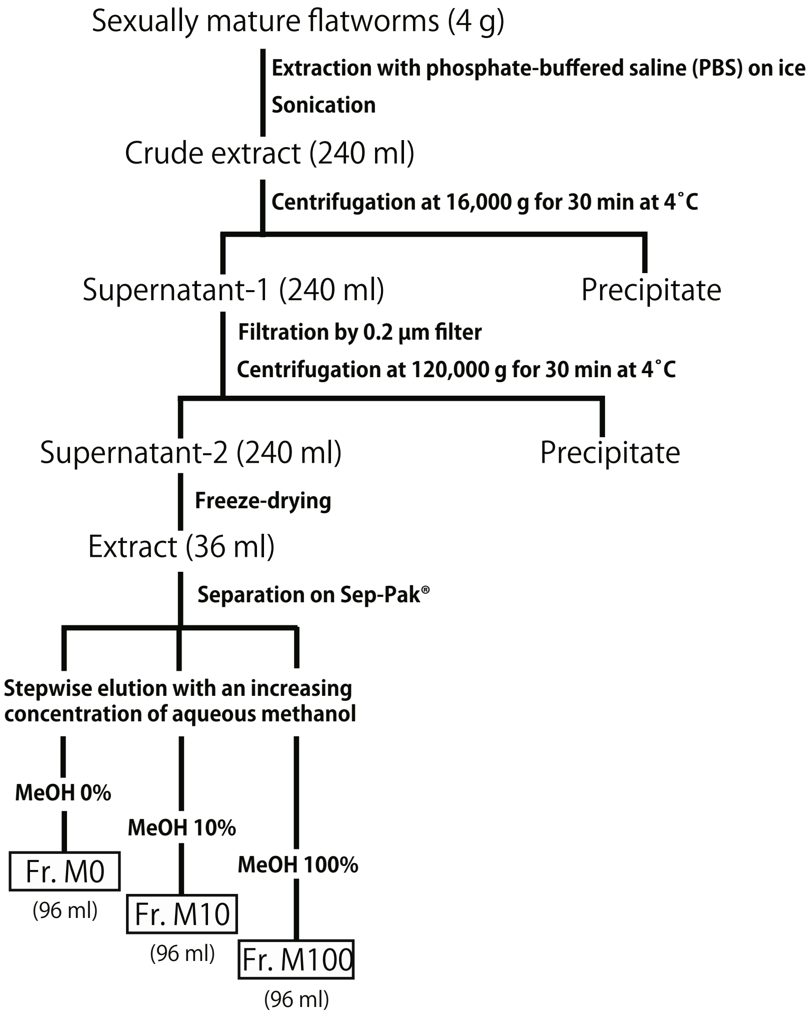

Figure S1. Fractionation procedure of biochemical substances contained in sexually mature flatworms using Sep-Pak® Light tC18 Cartridge; related to STAR Methods and Figures 2 and 3.

Fractionation was performed as shown in the diagram, allowing us to obtain Fr. M0, Fr. M10, and Fr. M100.

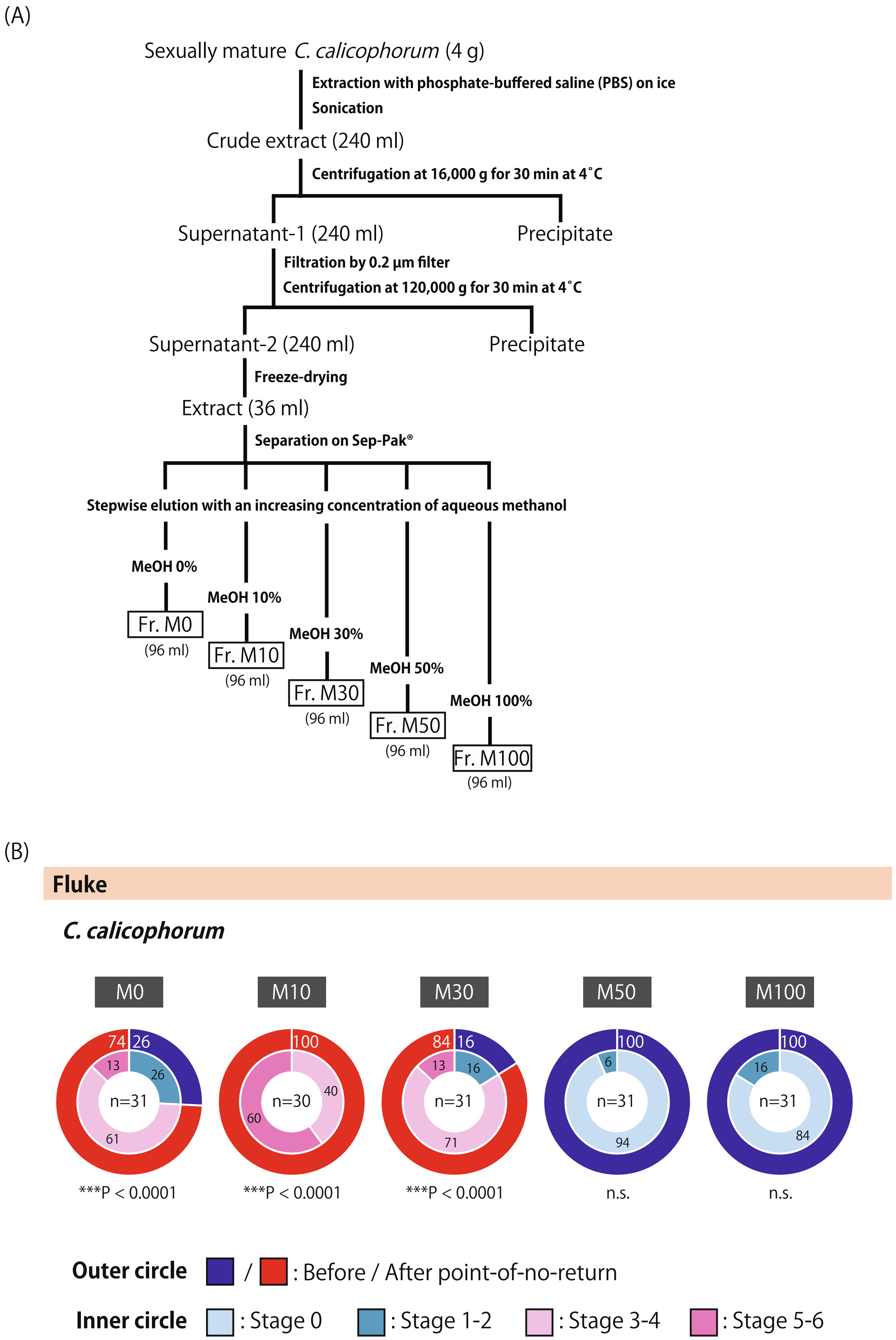
(A)
Sexually mature C. calicophorum (4 g)
Extraction with phosphate-buffered saline (PBS) on ice
Sonication
Crude extract (240 ml)
Centrifugation at 16,000 g for 30 min at 4˚C
Supernatant-1 (240 ml)
Precipitate
Filtration by 0.2 µm filter
Centrifugation at 120,000 g for 30 min at 4˚C
Supernatant-2 (240 ml)
Precipitate
Freeze-drying
Extract (36 ml)
Separation on Sep-Pak®
Stepwise elution with an increasing concentration of aqueous methanol
MeOH 0%
Fr. M0
(96 ml)
MeOH 10%
Fr. M10
(96 ml)
MeOH 30%
Fr. M30
(96 ml)
MeOH 50%
Fr. M50
(96 ml)
MeOH 100%
Fr. M100
(96 ml)
(B)
Fluke
C. calicophorum
M0
74
26
13
26
n=31
61
***P < 0.0001
M10
100
40
n=30
60
***P < 0.0001
M30
84
16
13
16
n=31
71
***P < 0.0001
M50
100
6
n=31
94
n.s.
M100
100
16
n=31
84
n.s.
Outer circle
/
: Before / After point-of-no-return
Inner circle
: Stage 0
: Stage 1-2
: Stage 3-4
: Stage 5-6

Figure S2. Additional feeding bioassay with five fractions from the fluke *Calicophoron calicophorum*; related to Figures 2 and 3.

(A) Fractionation using Sep-Pak® Light tC18 Cartridge was performed as shown in the diagram, using 4 g of sexually mature *C. calicophorum* (fluke). Fractions M0, M10, M30, M50, and M100 were mixed with chicken liver, and their sex-inducing effects on asexual *Dugesia ryukyuensis* worms were examined using a feeding bioassay for 4 weeks. **(B)** The percentages of worms in different developmental states are presented in doughnut charts; the outer circle shows the worms before and after the point of no-return, and the inner circle shows the sexualization stages of the worms. White and black numbers in the circles indicate percentages. Asterisks indicate significant differences in the number of worms before and after the point of no-return between the control and focal groups (Fisher's exact test: ***$P < 0.001$; n.s., not significant). Source data and statistics, including the exact *P*-values, are available in Supplemental Dataset 5. The sample size of each group is shown in the center of the doughnut chart.

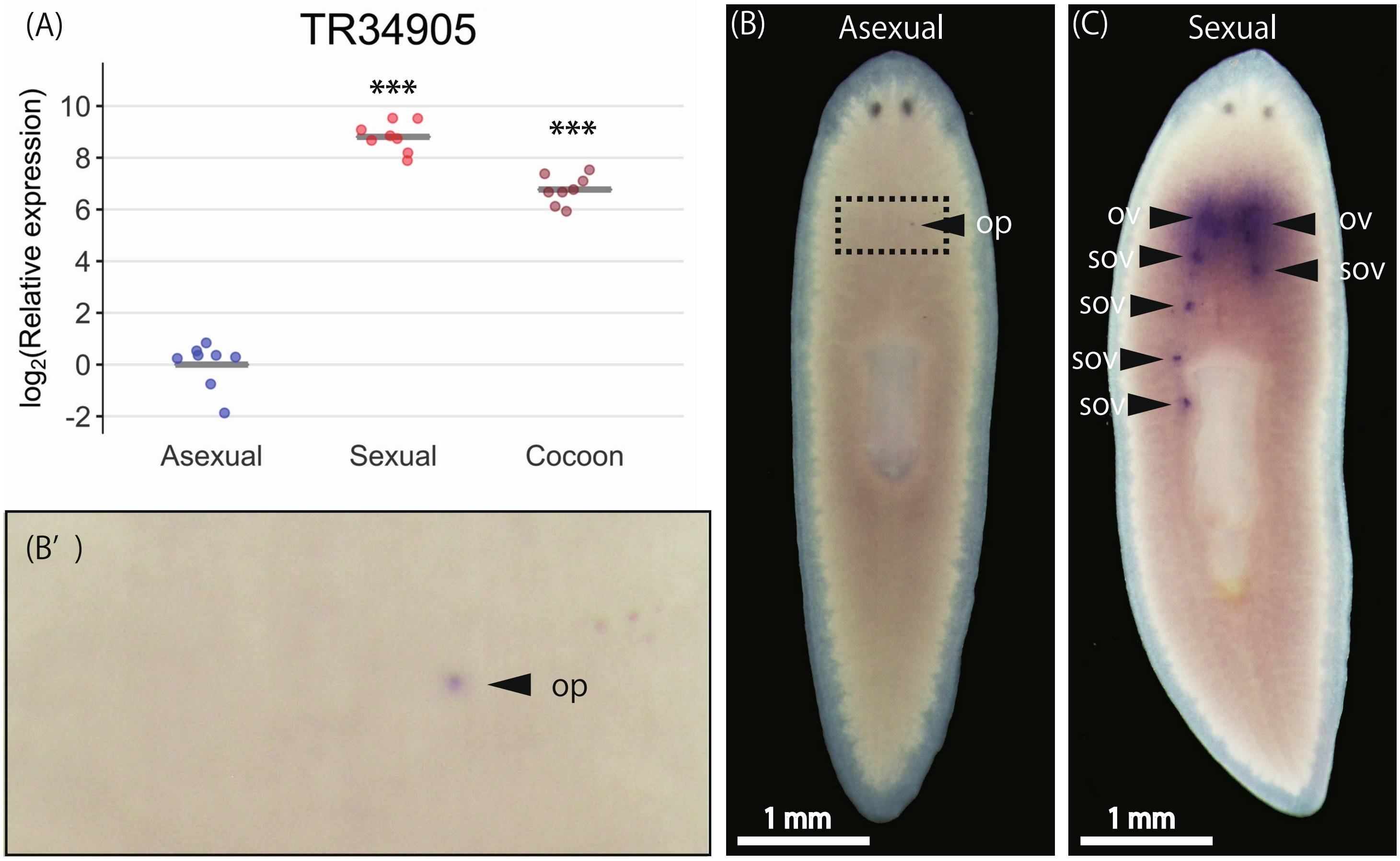

Figure S3. Isolation of the ovary marker gene *TR34905|c0_g1_i1* of *Dugesia ryukyuensis*; related to Figure 4.

The ovary marker gene *TR34905|c0_g1_i1* was first discovered during the annotation-based gene screening of a "*cgh*" homolog gene using the gene list provided in "Supplementary Dataset 3" of Sekii & Yorimoto et al. (2019) [1]. *Cgh* is an RNA helicase required during male and female gametogenesis in *Caenorhabditis elegans* [2]. After obtaining *TR34905|c0_g1_i1* and *TR37041|c0_g1_i1*, we observed that *TR34905|c0_g1_i1* expression was highly sexually biased (log_2 fold-change of 7.94 sexual/asexual) compared with that of *TR37041|c0_g1_i1* (log_2 fold-change of 1.86 sexual/asexual). Therefore, *TR34905|c0_g1_i1* was chosen for subsequent analyses. First, qRT-PCR analysis confirmed that *TR34905|c0_g1_i1* was highly expressed in sexual worms and the freshly laid cocoons (collected within 24 h of oviposition), compared with that in asexual worms (panel a). Whole-mount *in situ* hybridization analysis further revealed an ovary-specific gene expression pattern (panels B and C). In the asexual worms, a weak signal was occasionally detected in the ovarian primordium (1 out of 6 ovarian primordia [namely three pairs derived from three individual asexual worms]) (panels B and B', arrowhead with "op"). Moreover, in sexual worms, strong signals were detected from the main ovary (panel C, arrowhead with "ov") and the supernumerary ovaries (panel C, arrowhead with "sov"). Supernumerary ovaries are extra ectopic ovaries in addition to the main ovaries, which are often observed in experimentally sexualized *D. ryukyuensis* worms. No signal was detected from other tissues, such as the testis, vitellaria, or the copulatory organ. These results indicate that *TR34905|c0_g1_i1* is expressed from an early stage of oogonia to mature eggs, even after fertilization, demonstrating its validity as an ovary marker gene in the planarian *D. ryukyuensis*.

(A) The qRT-PCR data are shown relative to the expression level in the asexual worm, and log_2 (relative expression) on the vertical axis indicates $2^{-\Delta\Delta Ct}$. Each circle indicates an individual asexual worm, sexual worm, or cocoon. Eight replicates were used. The bars in the plots indicate the average $2^{-\Delta\Delta Ct}$ values. Asterisks indicate significant differences between the asexual worm, sexual worm, and freshly laid cocoon (Tukey's honestly significant difference test: ***$P < 0.001$). Source data and statistics, including the exact *P*-values, are available in Supplemental Dataset 5. **(B, C)** Representative gene expression patterns of *TR34905|c0_g1_i1* in **(B)** asexual and **(C)** sexual worms. Gene expression was observed as blue/purple staining (arrowhead). Images were taken from the ventral sides of the worms. The expression pattern was judged based on three replicates. **(B')** A high magnification of the dotted line box in B. Op, ovarian primordium; ov, ovary; sov, supernumerary ovary.

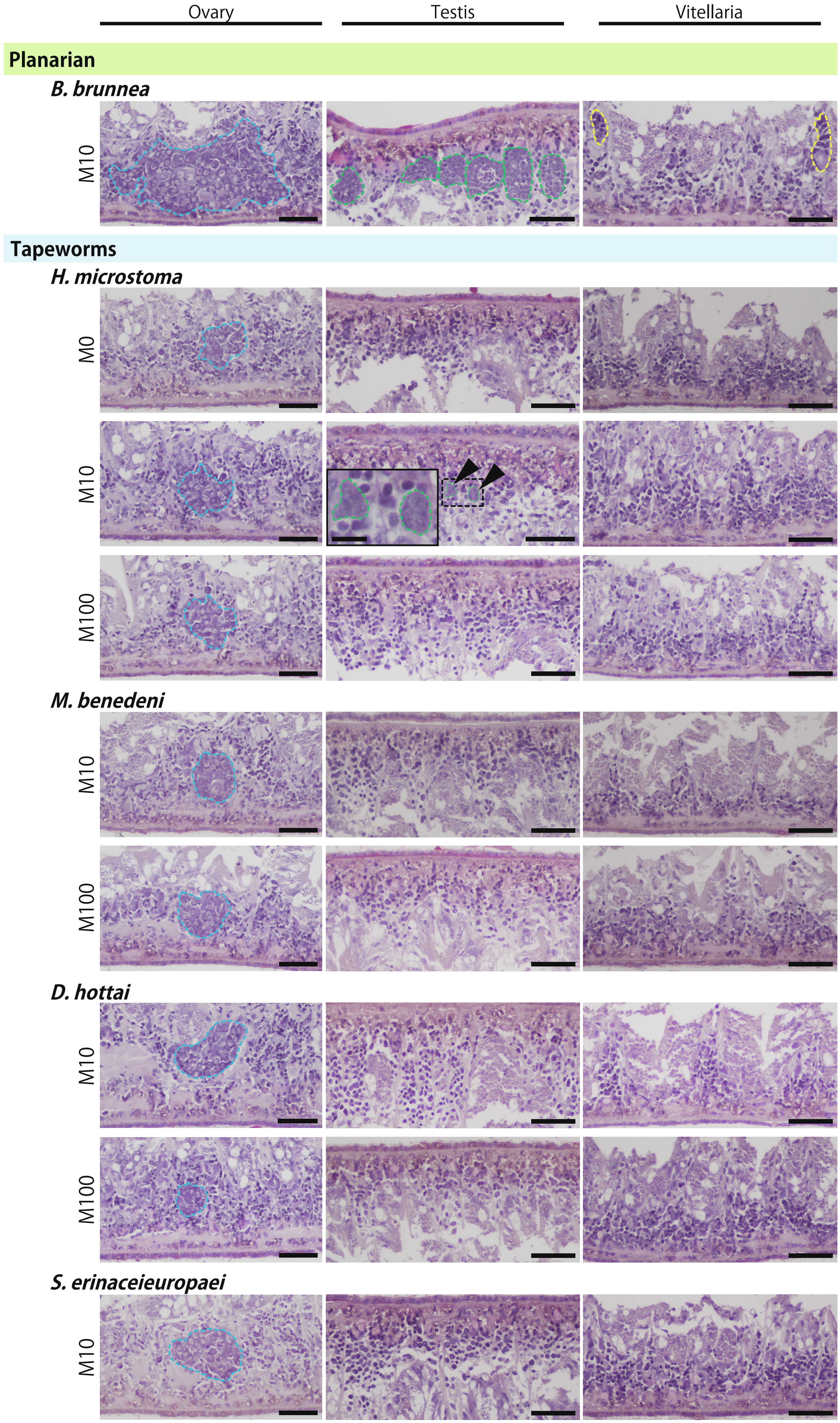

Ovary
Testis
Vitellaria
Planarian
B. brunnea
M10
Tapeworms
H. microstoma
M0
M10
M100
M. benedeni
M10
M100
D. hottai
M10
M100
S. erinaceieuropaei
M10

Figure S4. Histological evaluation of the sex-inducing effect of the fractions derived from tapeworms; related to Figure 4.

Although none of the tapeworm-derived fractions make the asexual *Dugesia ryukyuensis* worms go beyond the point of no-return (copulatory organs were not observed under the microscope), the development of reproductive organs was evaluated by histological sectioning for fractions with a statistically significant increase of testis and yolk gland markers in qRT-PCR (Fig. 4). The most sexually developed worm within each group fed with these fractions was sagittally sectioned and stained with hematoxylin and eosin (HE). The head sides are at the left, and the dorsal sides are at the top. Domains bound by the blue line are the female germ cell masses (ovaries); domains bound by the green line are the male germ cell masses (testes); domains bound by the yellow line are developing vitellaria. Arrowheads indicate two small testes found in the worm fed with Fr. M10 of *Hymenolepis microstoma*, and the solid line box is a high-magnification image of the dotted line box. Scale bars are 15 µm and 60 µm for the solid line box and others, respectively.

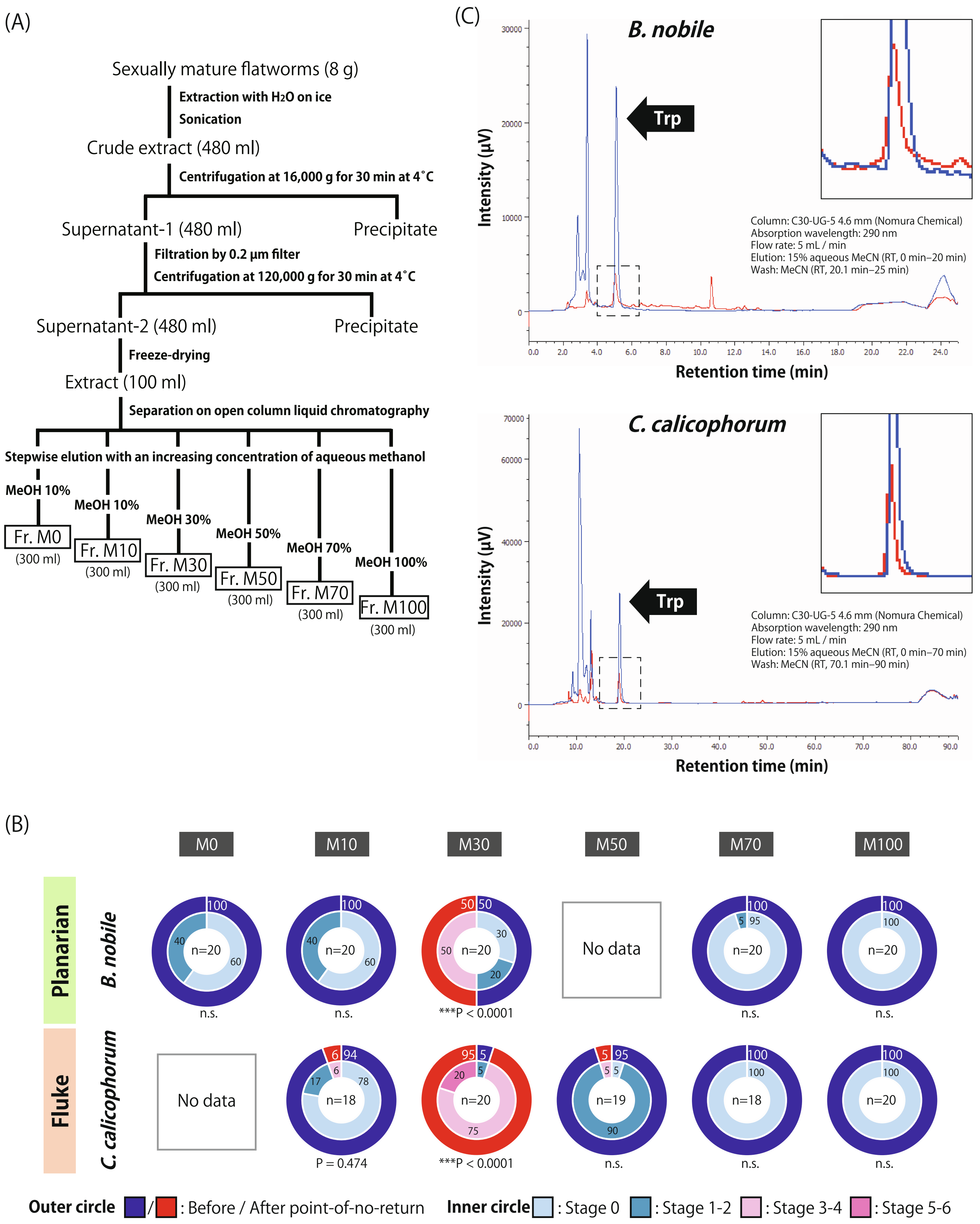

(A)
Sexually mature flatworms (8 g)
Extraction with H2O on ice
Sonication
Crude extract (480 ml)
Centrifugation at 16,000 g for 30 min at 4˚C
Supernatant-1 (480 ml)
Precipitate
Filtration by 0.2 µm filter
Centrifugation at 120,000 g for 30 min at 4˚C
Supernatant-2 (480 ml)
Precipitate
Freeze-drying
Extract (100 ml)
Separation on open column liquid chromatography
Stepwise elution with an increasing concentration of aqueous methanol
MeOH 10%
MeOH 10%
MeOH 30%
MeOH 50%
MeOH 70%
MeOH 100%
Fr. M0 (300 ml)
Fr. M10 (300 ml)
Fr. M30 (300 ml)
Fr. M50 (300 ml)
Fr. M70 (300 ml)
Fr. M100 (300 ml)
(B)
M0
M10
M30
M50
M70
M100
Planarian
B. nobile
Fluke
C. calicophorum
No data
n=20
n=18
n=19
n.s.
***P < 0.0001
P = 0.474
Outer circle : Before / After point-of-no-return
Inner circle : Stage 0 : Stage 1-2 : Stage 3-4 : Stage 5-6
(C)
B. nobile
C. calicophorum
Trp
Intensity (µV)
Retention time (min)
Column: C30-UG-5 4.6 mm (Nomura Chemical)
Absorption wavelength: 290 nm
Flow rate: 5 mL / min
Elution: 15% aqueous MeCN (RT, 0 min–20 min)
Wash: MeCN (RT, 20.1 min–25 min)
Column: C30-UG-5 4.6 mm (Nomura Chemical)
Absorption wavelength: 290 nm
Flow rate: 5 mL / min
Elution: 15% aqueous MeCN (RT, 0 min–70 min)
Wash: MeCN (RT, 70.1 min–90 min)

Figure S5. A new fractionation method for sex-inducing substances using open-column chromatography; related to STAR Methods and Figure 5.

(A) Fractionation was performed as shown in the diagram, using 8 g of sexually mature *Bipalium nobile* (planarian) or *C. calicophorum* (fluke). In the new method, extraction was done in water, and separation was conducted via open-column chromatography, yielding fractions (Fr.) M0, M10, M30, M50, M70, and M100. **(B)** The sex-inducing effects of Frs. M0, M10, M30, M50, M70, and M100 on asexual *Dugesia ryukyuensis* worms were examined using a feeding bioassay for 4 weeks. Samples derived from 4 g of *B. nobile* worms or 2 g of *C. calicophorum* worms were used in the feeding bioassays. The percentages of worms in different developmental states are presented in doughnut charts; the outer circle shows the worms before and after the point of no-return, and the inner circle shows the sexualization stages of the worms. White and black numbers in the circles indicate percentages. Asterisks indicate significant differences in the number of worms before and after the point of no-return between the control and focal groups (Fisher's exact test: ***$P < 0.001$; n.s., not significant). Source data and statistics, including the exact *P*-values, are available in Supplemental Dataset 5. The sample size of each group is shown in the center of the doughnut chart. **(C)** Differences in the quantities of tryptophan (Trp) contained in Frs. M10 and M30 were examined using reverse-phase high-performance liquid chromatography (HPLC). Samples derived from 0.016 g of flatworms were used for the analysis. The blue line indicates Fr. M10, and the red line indicates Fr. 30. The inset shows a high magnification of the dotted line box.

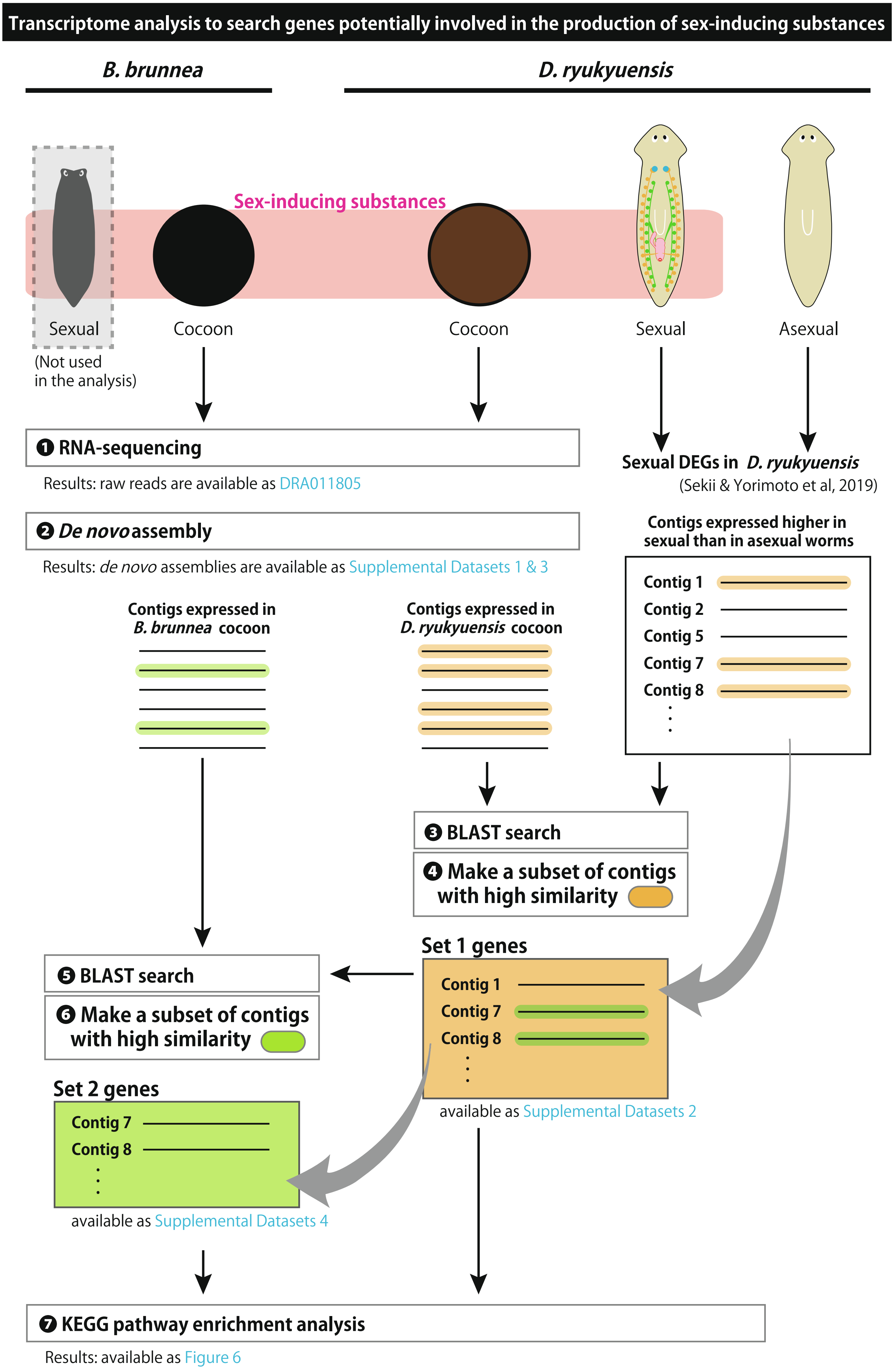

Transcriptome analysis to search genes potentially involved in the production of sex-inducing substances
B. brunnea
D. ryukyuensis
Sex-inducing substances
Sexual
(Not used in the analysis)
Cocoon
Cocoon
Sexual
Asexual
❶ RNA-sequencing
Results: raw reads are available as DRA011805
Sexual DEGs in D. ryukyuensis
(Sekii & Yorimoto et al, 2019)
❷ De novo assembly
Results: de novo assemblies are available as Supplemental Datasets 1 & 3
Contigs expressed higher in sexual than in asexual worms
Contig 1
Contig 2
Contig 5
Contig 7
Contig 8
Contigs expressed in B. brunnea cocoon
Contigs expressed in D. ryukyuensis cocoon
❸ BLAST search
❹ Make a subset of contigs with high similarity
Set 1 genes
Contig 1
Contig 7
Contig 8
available as Supplemental Datasets 2
❺ BLAST search
❻ Make a subset of contigs with high similarity
Set 2 genes
Contig 7
Contig 8
available as Supplemental Datasets 4
❼ KEGG pathway enrichment analysis
Results: available as Figure 6

Figure S6. Transcriptome analysis overview; related to STAR Methods and Figure 6.

Sex-inducing substances are contained in the sexual *Dugesia ryukyuensis* as well as *Bdellocephala brunnea* worms and cocoons but not in asexual worms. Transcriptome analysis was performed using the cocoons of *B. brunnea* and *D. ryukyuensis*. By combining the existing[1] and new RNA-seq datasets, candidate genes potentially involved in the production of sex-inducing substances in the planarian *D. ryukyuensis* were selected as follows. First, using information regarding the DEGs from Sekii & Yorimoto et al. (2019) [1], we obtained the sexual DEGs of the planarian *D. ryukyuensis* using the criteria of a false discovery rate (FDR) < 0.05 and $\log_2$ fold-change of sexual/asexual > 0. Using these sexual DEGs as query sequences, a Basic Local Alignment Search Tool (BLAST) search was performed against the *de novo* assembly transcript models of the planarian *D. ryukyuensis* cocoons. Sexual DEGs with similarity to the transcripts in *D. ryukyuensis* cocoons (with an e-value cutoff of e-120) were grouped as Set 1 genes. Next, using the Set 1 genes as query sequences, we performed a BLAST search against the *de novo* assembly transcript models of the planarian *B. brunnea* cocoons. Set 1 genes with similarity to the transcripts in *B. brunnea* cocoons (with an e-value cutoff of e-30) were grouped as Set 2 genes. Sets 1 and 2 were subjected to KEGG pathway enrichment analysis to identify the metabolic pathways enriched in the cocoons, which were expected to be associated with the production of conserved sex-inducing substances.

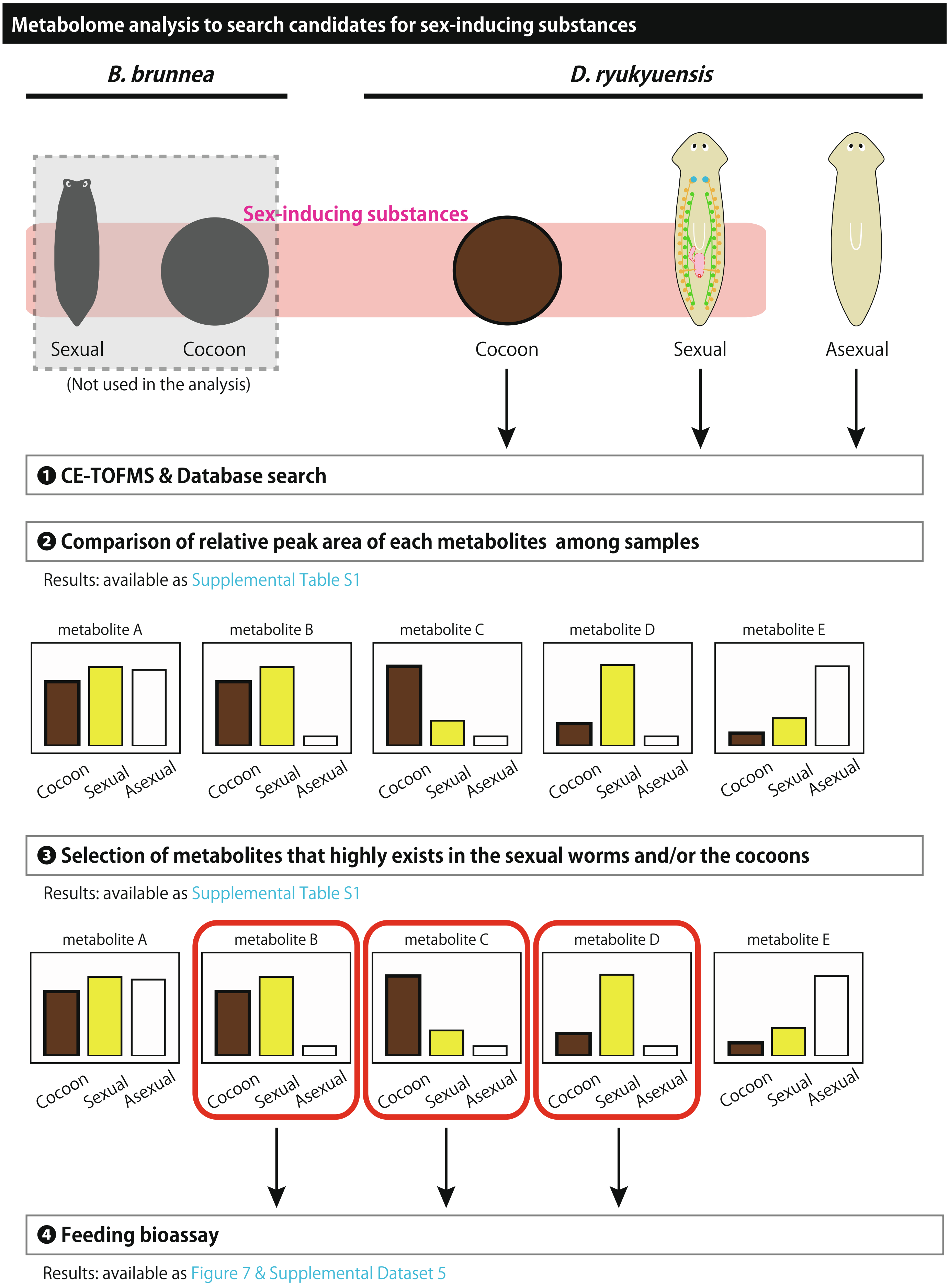

Metabolome analysis to search candidates for sex-inducing substances
B. brunnea
D. ryukyuensis
Sex-inducing substances
Sexual
Cocoon
(Not used in the analysis)
Cocoon
Sexual
Asexual
❶ CE-TOFMS & Database search
❷ Comparison of relative peak area of each metabolites among samples
Results: available as Supplemental Table S1
metabolite A
metabolite B
metabolite C
metabolite D
metabolite E
Cocoon
Sexual
Asexual
❸ Selection of metabolites that highly exists in the sexual worms and/or the cocoons
Results: available as Supplemental Table S1
metabolite A
metabolite B
metabolite C
metabolite D
metabolite E
Cocoon
Sexual
Asexual
❹ Feeding bioassay
Results: available as Figure 7 & Supplemental Dataset 5

Figure S7. Metabolome analysis overview; related to STAR Methods and Figures 6 and 7.

Sex-inducing substances are contained in the sexual *Dugesia ryukyuensis* and *Bdellocephala brunnea* worms and cocoons but not in asexual worms. Metabolome analysis was performed to compare the asexual *D. ryukyuensis* worms with sexual worms and cocoons. In this analysis, metabolites present in the HMT Inc. database (Tsuruoka, Japan) were identified. Note that unknown chemical compounds were not included. Metabolites present in the sexual worms and/or the cocoons at a concentration five times higher than that observed in asexual worms were selected as candidate sex-inducing substances. The sex-inducing effects of the identified candidates were tested using feeding bioassays.

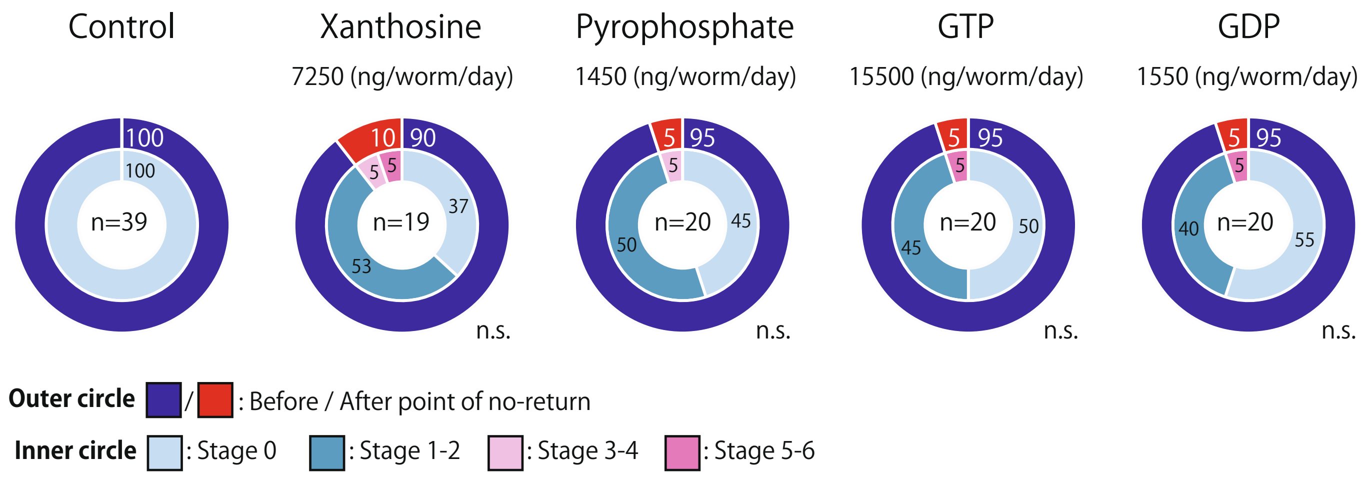

Figure S8. Feeding bioassay results for xanthosine, pyrophosphate, GTP, and GDP; related to Figure 7.

The percentages of worms in different developmental states after 4 weeks of metabolite-feeding bioassays are presented in doughnut charts; the outer circle shows the worms before and after the point of no-return, and the inner circle shows the sexualization stages of the worms. White and black numbers in the circles indicate percentage values. The number of worms before and after the point of no-return was not statistically different between the control and focal groups (Fisher's exact test, n.s., not significant). Source data and statistics, including the exact *P*-values, are available in Supplemental Dataset 5. The sample size of each group is shown in the center of the doughnut chart. The number of control worms is the sum of the control worms from several different bioassay batches.

Table S1. Metabolites detected in asexual worms, sexual worms, and cocoons; related to Figure 7. Metabolites were identified using the Human Metabolome Technologies database, based on the mass-to-charge ratio (m/z) and migration time (MT). Metabolites highly expressed in the sexual worms and/or the cocoons compared to that in the asexual worms (> 5:1 ratio) are indicated in pink. Compound names starting with “XA” and “XC” indicate metabolites detected in the anion and cation modes, respectively, but unidentified in the analysis. N.D., not detected; N.A., not available. Note that the number of replicates used in the metabolome analysis was n = 1, so no statistical analysis was applied for comparison. Asterisks indicate that the results were from Supplemental Table S1 of Kobayashi et al (2017)[3].

HMT DB		*m/z*	MT/RT	Relative area			Comparative analysis (ratio)		Feeding assay
Compound	HMDB ID			Asexuals	Sexuals	Cocoons	Sexuals vs Asexuals	Cocoons vs Asexuals	
Ser-Glu	No ID	235.093	10.54	N.D.	N.D.	5.6E-04	N.A.	1<	Examined
11-Aminoundecanoic acid	No ID	202.180	9.53	N.D.	N.D.	2.9E-04	N.A.	1<	Examined
3-Methoxytyramine	HMDB00022	168.102	8.69	N.D.	N.D.	2.1E-04	N.A.	1<	Examined
Mevalolactone	HMDB06024	131.070	22.34	N.D.	N.D.	2.8E-03	N.A.	1<	Examined
2-Phenylethylamine	HMDB12275	122.096	7.60	N.D.	N.D.	2.6E-03	N.A.	1<	Examined
Histamine	HMDB00870	112.087	4.64	N.D.	N.D.	3.4E-04	N.A.	1<	Examined
Homoserinelactone	No ID	102.054	6.97	N.D.	N.D.	1.5E-04	N.A.	1<	-
Ethanolamine	HMDB00149	62.061	6.21	N.D.	N.D.	3.3E-04	N.A.	1<	Examined
dTDP	HMDB01274	401.012	11.22	N.D.	N.D.	6.4E-05	N.A.	1<	Examined
Xanthosine	HMDB00299	283.068	7.55	N.D.	N.D.	7.5E-05	N.A.	1<	Examined
4-Pyridoxic acid	HMDB00017	182.047	8.67	N.D.	N.D.	7.6E-05	N.A.	1<	Examined
Homovanillic acid	HMDB00118	181.051	8.21	N.D.	N.D.	5.8E-05	N.A.	1<	Examined
Heptanoic acid	HMDB00666	129.092	8.53	N.D.	N.D.	5.7E-05	N.A.	1<	Examined
3-Hydroxybutyric acid	HMDB00011,HMDB00357,HM	103.040	9.49	N.D.	N.D.	6.7E-05	N.A.	1<	Examined
N-Methylproline	No ID	130.086	12.29	N.D.	7.3E-04	N.D.	1<	N.A.	Examined
Homoserine	HMDB00719	120.066	9.93	N.D.	3.1E-04	N.D.	1<	N.A.	Examined
Pyrophosphate	HMDB00250	176.937	16.52	N.D.	1.8E-03	N.D.	1<	N.A.	Examined
Terephthalic acid	HMDB02428	165.020	16.48	N.D.	4.6E-05	N.D.	1<	N.A.	Examined
4-Acetamidobutanoic acid	HMDB03681	144.068	8.40	N.D.	5.2E-05	N.D.	1<	N.A.	Examined
5-Hydroxytryptophan	HMDB00472	221.092	11.20	N.D.	1.7E-03	3.4E-02	1<	1<	Examined
XC0065	No ID	221.091	12.95	N.D.	4.1E-04	3.7E-04	1<	1<	-
3-Methoxytyrosine	HMDB01434	212.091	11.60	N.D.	3.3E-03	3.7E-03	1<	1<	Examined
Kynurenine	HMDB00684	209.090	9.75	N.D.	1.3E-04	4.1E-03	1<	1<	Examined
DOPA	HMDB00181,HMDB00609	198.075	11.52	N.D.	5.3E-03	4.3E-03	1<	1<	Examined
N-Methylglutamic acid	No ID	162.076	12.51	N.D.	6.4E-04	3.7E-04	1<	1<	Examined
$NADP^+$	HMDB00217	742.070	9.30	N.D.	3.6E-05	3.9E-04	1<	1<	Examined
3',5'-Cyclic dAMP	No ID	312.050	7.18	N.D.	2.7E-04	3.5E-05	1<	1<	Examined
Glucuronic acid	HMDB00127	193.036	8.02	N.D.	5.1E-05	9.5E-05	1<	1<	Examined
O-Phosphoserine	HMDB00272	184.003	12.22	N.D.	1.7E-04	2.3E-03	1<	1<	Examined
Ascorbic acid	HMDB00044	175.027	8.40	8.2E-05	2.4E-03	8.1E-04	29	10	Examined
Trp (*)	HMDB00929	205.096	10.84	1.4E-02	2.3E-01	6.1E-01	17	44	Examined
Tyr-Glu	No ID	311.123	10.95	6.0E-05	4.4E-04	2.3E-03	7.3	39	Examined
Tyr (*)	HMDB00158	182.080	11.19	2.2E-02	1.6E-01	6.5E-02	7.3	3.0	Examined
Taurocyamine	HMDB03584	168.043	22.12	2.9E-04	1.2E-03	2.3E-04	4.2	0.8	
Cysteine glutathione disulfide	HMDB00656	427.093	11.46	5.9E-04	2.2E-03	2.8E-04	3.7	0.5	-
2-Aminoethylphosphonic acid	HMDB11747	124.017	7.76	2.0E-04	7.2E-04	9.9E-04	3.6	5.0	-
His-Glu	No ID	285.117	7.42	6.0E-05	2.1E-04	2.7E-04	3.6	4.5	-
Cystathionine	HMDB00099	223.074	9.76	5.6E-03	2.0E-02	1.6E-02	3.5	2.9	-
XC0061	No ID	218.138	9.17	2.8E-04	9.0E-04	1.0E-03	3.2	3.7	-
UTP	HMDB00285	482.964	12.45	5.5E-04	1.6E-03	1.2E-03	2.8	2.2	-
Cys (*)	HMDB00574,HMDB03417	122.027	11.15	1.1E-03	3.0E-03	N.D.	2.8	<1	-
2-Aminoisobutyric acid<br>2-Aminobutyric acid	HMDB01906 HMDB00452	104.071	9.49	1.9E-03	5.0E-03	9.4E-04	2.6	0.5	-
Spermine	HMDB01256	203.221	4.34	1.8E-04	4.5E-04	3.9E-04	2.5	2.2	-
NAD^+	HMDB00902	662.104	6.48	3.9E-03	9.9E-03	4.3E-03	2.5	1.1	-
Lactic acid	HMDB00190,HMDB01311	89.025	10.70	4.4E-03	1.1E-02	9.5E-04	2.5	0.2	-
Succinic acid	HMDB00254	117.020	21.11	9.6E-04	2.4E-03	3.2E-03	2.5	3.3	-
6-Phosphogluconic acid	HMDB01316	275.018	14.63	2.8E-03	6.9E-03	2.3E-02	2.4	8.1	Examined
Betaine	HMDB00043	118.086	11.11	2.8E-02	6.6E-02	1.4E-01	2.3	4.9	-
UDP	HMDB00295	402.997	11.54	1.4E-03	3.2E-03	4.7E-03	2.3	3.3	-
GTP	HMDB01273	521.986	11.33	2.3E-04	5.0E-04	1.4E-03	2.2	6.3	Examined
3-Aminoisobutyric acid	HMDB03911	104.071	7.70	8.0E-03	1.7E-02	9.2E-03	2.2	1.1	-
N,*N*-Dimethylglycine	HMDB00092	104.071	10.77	1.0E-03	2.1E-03	2.3E-04	2.1	0.2	-
Glycerophosphocholine	HMDB00086	258.110	21.72	6.4E-03	1.4E-02	2.0E-02	2.1	3.1	-
β-Ala	HMDB00056	90.055	7.19	1.7E-03	3.4E-03	N.D.	2.1	<1	-
ATP	HMDB00538	505.990	11.65	4.8E-03	9.9E-03	6.9E-03	2.1	1.4	-
Sarcosine	HMDB00271	90.055	9.32	5.7E-04	1.1E-03	5.4E-04	2.0	1.0	-
Ethanolamine phosphate	HMDB00224	140.013	7.92	6.0E-04	1.1E-03	1.2E-03	1.9	2.1	-
CDP	HMDB01546	402.013	11.35	5.0E-05	9.3E-05	1.9E-04	1.9	3.7	-
Urocanic acid	HMDB00301	139.050	8.13	1.2E-02	2.2E-02	5.6E-04	1.8	0.0	-
Gln (*)	HMDB00641,HMDB03423	147.076	10.61	4.1E-02	7.3E-02	1.2E-02	1.8	0.3	-
Trimethylamine *N*-oxide	HMDB00925	76.076	6.60	2.0E-04	3.6E-04	2.0E-04	1.8	1.0	-
Glyceric acid	HMDB00139,HMDB06372	105.020	10.31	1.2E-04	2.0E-04	N.D.	1.7	<1	-

HMT DB		m/z	MT/RT	Relative area			Comparative analysis (ratio)		Feeding assay
Compound	HMDB ID			Asexuals	Sexuals	Cocoons	Sexuals vs Asexuals	Cocoons vs Asexuals	
Mevalonic acid	HMDB00227	147.067	8.52	1.3E-04	2.2E-04	1.6E-03	1.7	12.4	-
GDP	HMDB01201	442.018	10.51	6.0E-04	1.0E-03	5.0E-03	1.7	8.4	Examined
N-Acetylglutamic acid	HMDB01138	188.058	13.19	4.7E-03	7.9E-03	N.D.	1.7	<1	-
β-Ala-Lys	No ID	218.148	6.69	3.6E-04	5.9E-04	N.D.	1.7	<1	-
1-Methyl-4-imidazoleacetic acid	HMDB02820	141.065	8.15	2.4E-03	4.0E-03	N.D.	1.7	<1	-
ADP-glucose<br>GDP-fucose	HMDB06557 HMDB01095	588.077	8.24	7.8E-05	1.3E-04	N.D.	1.6	<1	-
Ophthalmic acid	HMDB05765	290.134	13.08	1.9E-04	3.1E-04	2.1E-04	1.6	1.1	-
3-Phosphoglyceric acid	HMDB00807	184.987	19.69	2.4E-03	3.9E-03	1.5E-03	1.6	0.6	-
Asp (*)	HMDB00191,HMDB06483	134.044	11.41	1.3E-01	2.0E-01	9.5E-02	1.6	0.7	-
Mucic acid	HMDB00639	209.031	14.06	1.9E-04	3.0E-04	1.4E-04	1.6	0.8	-
2-Phosphoglyceric acid	HMDB03391	184.987	19.20	3.2E-04	5.1E-04	2.3E-04	1.6	0.7	-
XC0071	No ID	234.181	6.08	1.1E-03	1.6E-03	5.3E-04	1.6	0.5	-
ADP	HMDB01341	426.024	10.78	1.1E-02	1.7E-02	2.0E-02	1.6	1.8	-
Phosphoenolpyruvic acid	HMDB00263	166.976	21.21	7.9E-04	1.2E-03	4.7E-04	1.5	0.6	-
Citric acid	HMDB00094	191.020	26.59	1.1E-03	1.6E-03	1.8E-03	1.5	1.6	-
S-Adenosylmethionine	HMDB01185	399.144	6.96	1.3E-03	2.0E-03	2.1E-03	1.5	1.6	-
Thiamine	HMDB00235	265.112	6.44	3.6E-05	5.4E-05	N.D.	1.5	<1	-
Phthalic acid	HMDB02107	165.020	15.38	1.3E-04	1.9E-04	N.D.	1.4	<1	-
Picolinic acid	HMDB02243	124.039	18.63	4.5E-03	6.4E-03	3.4E-03	1.4	0.8	-
Cytidine	HMDB00089	244.093	9.53	3.9E-03	5.5E-03	4.9E-04	1.4	0.1	-
5-Amino-4-oxovaleric acid	HMDB01149	132.066	7.86	9.8E-05	1.4E-04	N.D.	1.4	<1	-
Thr (*)	HMDB00167	120.065	10.37	2.6E-02	3.6E-02	4.2E-02	1.4	1.6	-
XC0126	No ID	310.112	14.84	8.3E-04	1.2E-03	4.9E-04	1.4	0.6	-
Spermidine	HMDB01257	146.165	4.40	4.0E-03	5.6E-03	5.2E-03	1.4	1.3	-
Malic acid	HMDB00156,HMDB00744	133.015	21.52	9.5E-03	1.3E-02	6.2E-03	1.4	0.7	-
Ala (*)	HMDB00161,HMDB01310	90.055	8.87	1.3E-01	1.8E-01	2.6E-02	1.4	0.2	-
Hydroxyproline	HMDB00725	132.066	11.88	3.2E-04	4.4E-04	2.2E-04	1.4	0.7	-
N-Glycolylneuraminic acid	HMDB00833	324.096	7.11	8.3E-03	1.1E-02	3.9E-03	1.4	0.5	-
Fumaric acid	HMDB00134	115.004	25.72	4.5E-04	6.1E-04	4.9E-04	1.4	1.1	-
XA0065	No ID	445.055	6.86	1.4E-03	1.8E-03	6.1E-05	1.3	0.0	-
UMP	HMDB00288	323.030	9.71	1.5E-03	2.0E-03	5.8E-03	1.3	3.9	-
O-Acetylcarnitine	HMDB00201	204.122	8.87	4.2E-03	5.6E-03	6.7E-04	1.3	0.2	-
Gluconic acid	HMDB00625	195.052	8.05	4.1E-04	5.4E-04	5.7E-04	1.3	1.4	-
ADP-ribose	HMDB01178	558.070	8.41	3.6E-05	4.7E-05	N.D.	1.3	<1	-
GDP-glucose	HMDB03351	604.071	8.21	2.4E-04	3.1E-04	9.8E-04	1.3	4.1	-
Carnitine	HMDB00062	162.112	8.36	1.4E-02	1.8E-02	5.8E-03	1.3	0.4	-
UDP-glucose<br>UDP-galactose	HMDB00286 HMDB00302	565.050	8.53	5.6E-03	7.2E-03	1.7E-03	1.3	0.3	-
Phosphorylcholine	HMDB01565	184.073	20.54	1.4E-02	1.8E-02	1.1E-02	1.3	0.8	-
dTDP-glucose	HMDB01328	563.069	8.36	1.5E-04	2.0E-04	7.1E-05	1.3	0.5	-
Asn (*)	HMDB00168	133.061	10.33	3.2E-02	4.1E-02	2.3E-02	1.3	0.7	-
N-Acetyllysine	HMDB00446	189.123	9.64	5.8E-04	7.2E-04	N.D.	1.2	<1	-
Pro (*)	HMDB00162,HMDB03411	116.071	10.65	2.4E-02	3.0E-02	1.2E-02	1.2	0.5	-
N-Acetylglucosamine	HMDB00215	222.099	22.24	5.1E-04	6.3E-04	N.D.	1.2	<1	-
XA0033	No ID	242.081	7.54	2.1E-03	2.6E-03	1.7E-03	1.2	0.8	-
Guanosine	HMDB00133	284.098	12.40	1.1E-03	1.4E-03	1.1E-04	1.2	0.1	-
IMP	HMDB00175	347.043	9.44	2.8E-04	3.4E-04	9.4E-05	1.2	0.3	-
Hexylamine	No ID	102.127	7.45	1.0E-03	1.2E-03	1.7E-03	1.2	1.7	-
CMP	HMDB00095	322.045	9.50	1.6E-04	1.9E-04	4.5E-04	1.2	2.9	-
Hypoxanthine	HMDB00157	137.046	10.99	8.0E-04	9.6E-04	5.3E-03	1.2	6.6	Examined
Rhein	No ID	283.024	8.54	8.2E-05	9.8E-05	N.D.	1.2	<1	-
N-Acetylornithine	HMDB03357	175.108	9.27	3.4E-04	4.1E-04	N.D.	1.2	<1	-
cAMP	HMDB00058	328.046	7.43	1.3E-04	1.6E-04	2.7E-05	1.2	0.2	-
p-Toluic acid	No ID	135.046	9.09	1.4E-04	1.6E-04	1.1E-04	1.2	0.8	-
Glutathione (GSSG)_divalent	HMDB03337	307.082	11.94	6.7E-02	7.8E-02	3.3E-02	1.2	0.5	-
FAD_divalent	HMDB01248	391.574	7.79	1.6E-04	1.8E-04	8.1E-05	1.2	0.5	-
Ile (*)	HMDB00172	132.101	10.06	6.6E-02	7.6E-02	1.7E-02	1.2	0.3	-
Carboxymethyllysine	No ID	205.117	9.06	8.4E-04	9.7E-04	4.2E-04	1.2	0.5	-
His (*)	HMDB00177	156.076	7.14	9.0E-02	1.0E-01	7.8E-02	1.1	0.9	-
GMP	HMDB01397	362.052	9.07	1.3E-03	1.5E-03	1.5E-03	1.1	1.2	-
S-Adenosylhomocysteine	HMDB00939	385.126	8.58	2.1E-04	2.4E-04	7.6E-04	1.1	3.6	-
GABA	HMDB00112	104.071	7.54	3.9E-03	4.4E-03	3.8E-04	1.1	0.1	-
Thiamine diphosphate	HMDB01372	423.031	7.86	7.9E-05	8.9E-05	3.9E-05	1.1	0.5	-
N-Acetylalanine	HMDB00766	130.051	8.81	4.7E-05	5.3E-05	N.D.	1.1	<1	-
Putrescine	HMDB01414	89.108	4.56	9.6E-02	1.1E-01	7.4E-02	1.1	0.8	-
CDP-choline	HMDB01413	487.102	6.71	7.8E-04	8.6E-04	1.3E-03	1.1	1.7	-
Octanoic acid	HMDB00482	143.108	8.28	1.7E-04	1.8E-04	9.9E-05	1.1	0.6	-
CMP-*N*-acetylneuraminate	HMDB01176	613.145	8.22	2.1E-03	2.4E-03	1.1E-03	1.1	0.5	-
γ-Glu-Cys	HMDB01049	251.069	12.67	8.6E-04	9.4E-04	1.4E-04	1.1	0.2	-

HMT DB		*m/z*	MT/RT	Relative area			Comparative analysis (ratio)		Feeding assay
Compound	HMDB ID			Asexuals	Sexuals	Cocoons	Sexuals vs Asexuals	Cocoons vs Asexuals	
UDP-*N*-acetylglucosamine	HMDB00290	606.076	8.36	1.8E-02	2.0E-02	8.7E-03	1.1	0.5	-
myo-Inositol 2-phosphate	No ID	259.024	10.39	5.8E-03	6.2E-03	4.9E-04	1.1	0.1	-
Ornithine	HMDB00214,HMDB03374	133.097	6.67	2.7E-02	2.9E-02	1.2E-03	1.1	0.0	-
Methionine sulfoxide	HMDB02005	166.053	11.72	1.5E-03	1.6E-03	5.6E-04	1.1	0.4	-
Phe (*)	HMDB00159	166.085	10.92	4.8E-02	5.2E-02	8.1E-02	1.1	1.7	-
Lys (*)	HMDB00182,HMDB03405	147.112	6.72	8.9E-02	9.4E-02	4.6E-02	1.1	0.5	-
Gly-Leu	No ID	189.123	9.56	1.2E-03	1.3E-03	N.D.	1.1	<1	-
Pyridoxamine 5'-phosphate	HMDB01555	249.066	10.30	3.5E-04	3.8E-04	8.6E-05	1.1	0.2	-
Cysteic acid	HMDB02757	167.997	11.03	1.0E-04	1.1E-04	N.D.	1.1	<1	-
Tyramine	HMDB00306	138.091	8.12	2.6E-04	2.8E-04	1.2E-01	1.1	450.9	Examined
2,4-Diaminobutyric acid	HMDB06284	119.082	6.75	2.1E-04	2.2E-04	N.D.	1.1	<1	-
XC0089	No ID	255.097	9.39	7.2E-03	7.6E-03	7.7E-03	1.1	1.1	-
2-Aminoadipic acid	HMDB00510	162.076	10.79	2.0E-03	2.1E-03	8.2E-04	1.1	0.4	-
FMN	HMDB01520	455.100	8.27	5.5E-05	5.8E-05	N.D.	1.0	<1	-
N-Acetylglucosamine 6-phosphate	HMDB01062	300.051	9.09	2.1E-03	2.2E-03	N.D.	1.0	<1	-
Fructose 1,6-diphosphate	HMDB01058	338.990	14.53	8.4E-04	8.7E-04	7.5E-04	1.0	0.9	-
Ser (*)	HMDB00187,HMDB03406	106.050	9.84	5.5E-02	5.7E-02	3.5E-02	1.0	0.6	-
Gly (*)	HMDB00123	76.039	8.17	2.1E-02	2.2E-02	6.1E-03	1.0	0.3	-
3-Methylhistidine	HMDB00479	170.092	7.34	6.0E-03	6.2E-03	1.6E-03	1.0	0.3	-
Serotonin	HMDB00259	177.102	8.60	7.4E-03	7.6E-03	N.D.	1.0	<1	-
myo-Inositol 1-phosphate<br>*myo*-Inositol 3-phosphate	HMDB00213 HMDB06814	259.024	10.21	7.9E-03	8.0E-03	4.3E-04	1.0	0.1	-
Ala-Ala	HMDB03459	161.091	9.11	5.1E-04	5.2E-04	N.D.	1.0	<1	-
Guanine	HMDB00132	152.057	8.16	1.3E-02	1.3E-02	6.9E-03	1.0	0.5	-
Fructose 6-phosphate	HMDB00124	259.024	9.88	6.4E-03	6.5E-03	7.2E-04	1.0	0.1	-
Leu (*)	HMDB00687	132.101	10.16	9.1E-02	9.2E-02	1.8E-02	1.0	0.2	-
Glutathione (GSH)	HMDB00125	308.090	13.03	8.6E-02	8.7E-02	1.8E-02	1.0	0.2	-
Saccharopine	HMDB00279	277.139	10.58	1.1E-02	1.1E-02	3.9E-03	1.0	0.4	-
Glucose 6-phosphate	HMDB01401	259.024	9.78	2.5E-02	2.5E-02	1.4E-03	1.0	0.1	-
N-Acetylneuraminic acid	HMDB00230	308.100	7.13	6.3E-03	6.3E-03	1.9E-03	1.0	0.3	-
Sedoheptulose 7-phosphate	HMDB01068	289.034	9.54	1.1E-02	1.1E-02	1.6E-03	1.0	0.1	-
Decanoic acid	HMDB00511	171.140	7.88	1.4E-04	1.4E-04	8.4E-05	1.0	0.6	-
Choline	HMDB00097	104.107	6.71	1.6E-02	1.6E-02	2.8E-03	1.0	0.2	-
Pelargonic acid	HMDB00847	157.124	8.05	6.3E-04	6.2E-04	4.4E-04	1.0	0.7	-
Val (*)	HMDB00883	118.086	9.86	8.3E-02	8.1E-02	1.5E-02	1.0	0.2	-
γ-Butyrobetaine	HMDB01161	146.117	7.94	5.9E-04	5.7E-04	N.D.	1.0	<1	-
3-Hydroxyanthranilic acid	HMDB01476	154.050	10.75	1.4E-04	1.4E-04	1.9E-04	1.0	1.3	-
N^2-Acetylaminoadipic acid	No ID	202.073	12.36	3.5E-04	3.3E-04	N.D.	0.9	<1	-
Adenine	HMDB00034	136.061	7.48	1.3E-04	1.3E-04	9.1E-05	0.9	0.7	-
N-Acetylglucosamine 1-phosphate	HMDB01367	300.051	9.47	2.7E-03	2.5E-03	9.7E-05	0.9	0.0	-
Glucose 1-phosphate	HMDB01586	259.024	10.07	2.4E-03	2.3E-03	2.0E-04	0.9	0.1	-
Riboflavin	HMDB00244	377.143	22.02	6.1E-03	5.7E-03	3.2E-03	0.9	0.5	-
Inosine	HMDB00195	269.087	19.55	3.5E-04	3.3E-04	N.D.	0.9	<1	-
Acetylcholine	HMDB00895	146.117	7.46	7.2E-03	6.7E-03	N.D.	0.9	<1	-
Diethanolamine	HMDB04437	106.086	7.50	1.0E-03	9.3E-04	1.4E-03	0.9	1.4	-
Lauric acid	HMDB00638	199.172	7.58	9.3E-04	8.6E-04	1.1E-04	0.9	0.1	-
Hexanoic acid	HMDB00535	115.077	8.85	8.0E-05	7.4E-05	N.D.	0.9	<1	-
Homocitrulline	HMDB00679	190.118	11.02	5.1E-04	4.7E-04	N.D.	0.9	<1	-
3-Ureidopropionic acid	HMDB00026	131.047	9.21	6.7E-04	6.1E-04	N.D.	0.9	<1	-
XC0132	No ID	325.160	8.52	5.4E-04	4.9E-04	8.9E-05	0.9	0.2	-
Xanthine	HMDB00292	151.025	8.21	4.2E-04	3.8E-04	8.0E-05	0.9	0.2	-
Arg (*)	HMDB00517,HMDB03416	175.118	6.96	8.5E-02	7.6E-02	2.4E-02	0.9	0.3	-
Ribose 5-phosphate	HMDB01548	229.013	10.48	3.1E-04	2.8E-04	6.3E-05	0.9	0.2	-
AMP	HMDB00045	346.057	9.26	2.8E-02	2.5E-02	2.7E-02	0.9	1.0	-
Nicotinamide	HMDB01406	123.055	7.25	9.5E-04	8.4E-04	N.D.	0.9	<1	-
Gly-Asp	No ID	191.067	9.73	2.5E-04	2.2E-04	2.6E-04	0.9	1.1	-
Adenosine	HMDB00050	268.103	9.72	1.0E-03	8.5E-04	7.4E-04	0.9	0.7	-
5'-Deoxy-5'-methylthioadenosine	HMDB01173	298.097	9.94	1.3E-04	1.1E-04	2.1E-04	0.9	1.7	-
Agmatine	HMDB01432	131.129	5.03	1.3E-03	1.1E-03	2.8E-04	0.9	0.2	-
N^5-Ethylglutamine	No ID	175.107	11.20	6.4E-04	5.5E-04	N.D.	0.8	<1	-
Azelaic acid	HMDB00784	187.099	12.09	1.8E-04	1.5E-04	N.D.	0.8	<1	-
Thymidine	HMDB00273	243.096	22.16	5.1E-04	4.2E-04	3.0E-04	0.8	0.6	-
Glu (*)	HMDB00148,HMDB03339	148.060	10.78	2.4E-01	1.9E-01	2.7E-02	0.8	0.1	-
N^6-Acetyllysine	HMDB00206	189.123	11.28	7.2E-04	5.7E-04	N.D.	0.8	<1	-
Cytosine	HMDB00630	112.051	7.10	9.6E-05	7.6E-05	N.D.	0.8	<1	-
Cadaverine	HMDB02322	103.123	4.85	1.1E-02	8.6E-03	1.1E-03	0.8	0.1	-
5-Oxoproline	HMDB00267	128.036	9.42	3.8E-04	3.0E-04	2.2E-04	0.8	0.6	-
Ribulose 5-phosphate	HMDB00618	229.013	10.85	1.5E-03	1.1E-03	1.6E-04	0.8	0.1	-
Uridine	HMDB00296	245.076	22.21	2.1E-02	1.6E-02	N.D.	0.8	<1	-
Pantothenic acid	HMDB00210	218.105	7.59	1.3E-03	9.7E-04	5.9E-04	0.8	0.5	-

HMT DB		m/z	MT/RT	Relative area			Comparative analysis (ratio)		Feeding assay
Compound	HMDB ID			Asexuals	Sexuals	Cocoons	Sexuals vs Asexuals	Cocoons vs Asexuals	
3-Phenylpropionic acid	HMDB00764	149.061	8.73	2.5E-04	1.9E-04	9.5E-05	0.8	0.4	-
Octopamine<br>Dopamine	HMDB04825 HMDB00073	154.086	8.52	2.1E-03	1.6E-03	1.7E-02	0.8	8.2	Examined
Uracil	HMDB00300	113.034	22.16	2.6E-03	1.9E-03	N.D.	0.7	<1	-
N_{ω}-Methylarginine	No ID	189.134	7.31	7.7E-04	5.6E-04	1.4E-04	0.7	0.2	-
Dyphylline	No ID	255.106	22.22	3.4E-03	2.5E-03	3.1E-04	0.7	0.1	-
Taurocholic acid	HMDB00036	514.286	6.64	9.3E-05	6.7E-05	N.D.	0.7	<1	-
N^6-Methyllysine	HMDB02038	161.128	6.94	9.2E-04	6.6E-04	2.5E-04	0.7	0.3	-
SDMA	HMDB03334	203.150	7.64	1.3E-03	9.3E-04	3.3E-04	0.7	0.2	-
Glycerol	HMDB00131	93.055	22.16	1.4E-01	9.7E-02	1.6E-01	0.7	1.1	-
Dihydroxyacetone phosphate	HMDB01473	168.992	12.61	2.0E-03	1.4E-03	6.7E-05	0.7	0.0	-
N^6,N^6,N^6-Trimethyllysine	HMDB01325	189.158	7.03	2.4E-03	1.6E-03	3.6E-04	0.7	0.2	-
Nicotinic acid	HMDB01488	124.039	9.85	8.7E-04	5.6E-04	5.8E-04	0.7	0.7	-
Glycerol 3-phosphate	HMDB00126	171.006	12.06	3.8E-03	2.4E-03	8.0E-04	0.6	0.2	-
Citrulline	HMDB00904	176.102	10.89	1.9E-02	1.2E-02	7.1E-03	0.6	0.4	-
Met (*)	HMDB00696	150.058	10.58	1.6E-02	9.3E-03	1.6E-03	0.6	0.1	-
N-Acetylputrescine	HMDB02064	131.118	8.30	6.3E-04	3.5E-04	N.D.	0.6	<1	-
Triethanolamine	No ID	150.112	8.09	5.7E-04	3.2E-04	3.8E-04	0.6	0.7	-
NMN	HMDB00229	335.063	20.66	1.5E-02	8.3E-03	5.6E-04	0.5	0.0	-
ADMA	HMDB01539	203.150	7.51	8.4E-03	4.6E-03	8.1E-04	0.5	0.1	-
Argininosuccinic acid	HMDB00052	291.129	9.27	1.0E-03	5.4E-04	2.0E-04	0.5	0.2	-
Trigonelline	HMDB00875	138.056	10.30	5.9E-03	2.8E-03	1.8E-04	0.5	0.0	-
Pipecolic acid	HMDB00070,HMDB00716,HM	130.086	10.12	1.8E-02	8.2E-03	6.5E-04	0.5	0.0	-
7-Methylguanine	HMDB00897	166.073	8.09	4.1E-04	1.7E-04	N.D.	0.4	<1	-
Uric acid	HMDB00289	167.021	8.81	1.8E-04	7.1E-05	4.0E-05	0.4	0.2	-
N^8-Acetylspermidine	HMDB02189	188.175	6.19	3.3E-04	1.1E-04	N.D.	0.3	<1	-
3-Methyladenine	HMDB11600	150.076	7.78	1.1E-03	3.1E-04	6.3E-05	0.3	0.1	-
3-Methylguanine	HMDB01566	166.072	8.33	1.9E-04	N.D.	N.D.	<1	<1	-
4-Guanidinobutyric acid	HMDB03464	146.092	8.14	2.3E-04	N.D.	N.D.	<1	<1	-
N-Acetyltryptophan	No ID	245.095	7.65	5.5E-05	N.D.	4.9E-05	<1	0.9	-

Table S2. List of chemical compounds used for the feeding bioassays; related to STAR Methods and Figure 7.

Compound	Product name	Manufacturer	Catalog No.
11-Aminoundecanoic acid	11-Aminoundecanoic acid	Ark Pharm (USA)	AK111174
2-Phenylethylamine	2-Phenylethylamine hydrochroride	Sigma-Aldrich (USA)	P6513-25G
3-Hydroxybutyric acid	DL-3-Hydroxybutyric Acid	Tokyo Chemical Industry (Japan)	H0228
3-Methoxytyramine	3-METHOXYTYRAMINE HYDROCHLORIDE	MP Biomedicals (USA)	105665
3-Methoxytyrosine	3-O-Methyl-L-DOPA Monohydrate	Toront Reserarch Chemicals (Canada)	M303815
3',5'-Cyclic dAMP	2'-Deoxyadenosine 3':5'-cyclic monophosphate sodium salt	Santa Cruz Biotechnology (USA)	sc-214051
4-Acetamidobutanoic acid	4-Acetamidobutyric acid	Santa Cruz Biotechnology (USA)	sc-276980
4-Pyridoxic acid	4-Pyridoxic acid	Sigma-Aldrich (USA)	P9630-25MG
5-Hydroxytryptophan	5-Hydroxy-L-Tryptophan	Sigma-Aldrich (USA)	107751-1G
Ascorbic acid	L(+)-Ascorbic Acid	nacalai tesque (Japan)	03420-52
DOPA	L-DOPA	nacalai tesque (Japan)	14211-81
Dopamine	Dopamine Hydrochloride	nacalai tesque (Japan)	14212-71
dTDP	Thymidine 5'-diphosphate sodium salt	Cosmo Bio (Japan)	sc-215980
Ethanolamine	2-Aminoethanol	Wako (Japan)	016-12453
GDP	Guanosine 5'-diphosphate sodium salt	Sigma-Aldrich (USA)	G7127-25MG
Glucuronic acid	D-Glucuronic acid	Sigma-Aldrich (USA)	G5269-10G
GTP	Guanosine 5'-triphosphate sodium salt	Sigma-Aldrich (USA)	G8877-25MG
Heptanoic acid	Heptanoic Acid	nacalai tesque (Japan)	14316-92
Histamine	Histamine (free base)	nacalai tesque (Japan)	18111-71
Homovanillic acid	Homovanillic acid Fluorimetric reagent	Sigma-Aldrich (USA)	H1252-100MG
Hypoxanthine	Hypoxanthine	nacalai tesque (Japan)	17487-01
Kynurenine	L-Kynurenine	Sigma-Aldrich (USA)	K8625-25MG
L-Homoserine	L-Homoserine	Sigma-Aldrich (USA)	H1030
N-Methylglutamic acid	N-Me-Glu-OH	bachem (Switzerland)	4002660.0001
N-Methylproline	(S)-1-Methylpyrrolidine-2-carboxylic acid hydrate	Ark Pharm (USA)	AK-46644
NADP+	NADP, disodium salt, approx. 98%	Roche (Switzerland)	10128031001
O-Phosphoserine	O-Phospho-L-serine	nacalai tesque (Japan)	27833-41
Octopamine	(±)-Octopamine hydrochloride	Sigma-Aldrich (USA)	O0250-1G
Pyrophosphate	Sodium pyrophosphate tetrabasic decahydrate	Sigma-Aldrich (USA)	S6422-100G
Mevalolactone	(±)-Mevalonolactone	Sigma-Aldrich (USA)	M4667-1G
Ser-Glu	Ser-Glu	AnaSpec Inc	AS-65126-SE
Terephthalic acid	Terephthalic acid	Sigma-Aldrich (USA)	185361-5G
Tyr-Glu	Tyr-Glu	AnaSpec Inc	65126-YE
Tyramine	Tyramine	MP Biomedicals (USA)	A0302
Xanthosine	Xanthosine dihydrate	Sigma-Aldrich (USA)	X0750-5G
Tyr	L-Tyrosine	nacalai tesque (Japan)	35709-44
6-Phosphogluconic acid	6-Phospho-D-Gluconate, trisodium salt	Wako (Japan)	45190000

References

1. Sekii, K., Yorimoto, S., Okamoto, H., Nagao, N., Maezawa, T., Matsui, Y., Yamaguchi, K., Furukawa, R., Shigenobu, S., and Kobayashi, K. (2019). Transcriptomic analysis reveals differences in the regulation of amino acid metabolism in asexual and sexual planarians. Sci. Rep. *9*. https://doi.org/10.1038/s41598-019-42025-z.

2. Navarro, R.E., Shim, E.Y., Kohara, Y., Singson, A., and Blackwell, T.K. (2001). *cgh-1*, a conserved predicted RNA helicase required for gametogenesis and protection from physiological germline apoptosis in *C. elegans*. Development *128*, 3221-3232.

3. Kobayashi, K., Maezawa, T., Tanaka, H., Onuki, H., Horiguchi, Y., Hirota, H., Ishida, T., Horiike, K., Agata, Y., Aoki, M., et al. (2017). The identification of D-tryptophan as a bioactive substance for postembryonic ovarian development in the planarian *Dugesia ryukyuensis*. Sci. Rep. *7*, 45175. 10.1038/srep45175.
